# Hybrid Triboelectric-Electromagnetic Nanogenerators for Mechanical Energy Harvesting: A Review

**DOI:** 10.1007/s40820-021-00713-4

**Published:** 2021-09-20

**Authors:** João V. Vidal, Vladislav Slabov, Andrei L. Kholkin, Marco P. Soares dos Santos

**Affiliations:** 1 Department of Physics and CICECO – Aveiro Institute of Materials, University of Aveiro, 3810-193 Aveiro, Portugal; 2 Department of Physics and I3N, University of Aveiro, 3810-193 Aveiro, Portugal; 3 Laboratory of Functional Low-Dimensional Structures, National University of Science and Technology MISIS, Moscow, Russia; 4 Department of Mechanical Engineering, Centre for Mechanical Technology and Automation (TEMA), University of Aveiro, 3810-193 Aveiro, Portugal

**Keywords:** E-TENG, Hybrid triboelectric-electromagnetic, Nanogenerators, Energy harvesting

## Abstract

Motion-driven electromagnetic-triboelectric energy generators (E-TENGs) hold a great potential to provide higher voltages, higher currents and wider operating bandwidths than both electromagnetic and triboelectric generators standing alone. Therefore, they are promising solutions to autonomously supply a broad range of highly sophisticated devices. This paper provides a thorough review focused on major recent breakthroughs in the area of electromagnetic-triboelectric vibrational energy harvesting. A detailed analysis was conducted on various architectures including rotational, pendulum, linear, sliding, cantilever, flexible blade, multidimensional and magnetoelectric, and the following hybrid technologies. They enable highly efficient ways to harvest electric energy from many forms of vibrational, rotational, biomechanical, wave, wind and thermal sources, among others. Open-circuit voltages up to 75 V, short-circuit currents up to 60 mA and instantaneous power up to 144 mW were already achieved by these nanogenerators. Their transduction mechanisms, including proposed models to make intelligible the involved physical phenomena, are also overviewed here. A comprehensive analysis was performed to compare their respective construction designs, external excitations and electric outputs. The results highlight the potential of hybrid E-TENGs to convert unused mechanical motion into electric energy for both large- and small-scale applications. Finally, this paper proposes future research directions toward optimization of energy conversion efficiency, power management, durability and stability, packaging, energy storage, operation input, research of transduction mechanisms, quantitative standardization, system integration, miniaturization and multi-energy hybrid cells.

## Highlights

- An up-to-date review of hybrid triboelectric-electromagnetic nanogenerators is provided.
- Rotational, pendulum, linear, sliding, cantilever, flexible blade, multidimensional, and magnetoelectric hybrid technologies are thoroughly analyzed.
- Promising results highlight the potential of these hybrid technologies for both small-scale and large-scale powering.

## Graphical abstract

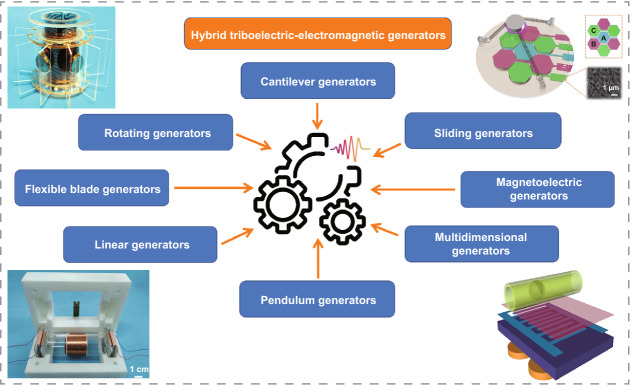

## Introduction

Energy is one of the most important resources with a huge demand from the part of modern society due to its central standing to the general quality of life [1]. The energy transition may be one of the greatest challenges facing humanity today driven by environmental and limited fossil fuel concerns. Thus, the demand for new large-scale sustainable forms of energy, such as solar, wind and hydroelectric, is enormous. The near future is further expected to see a worldwide distribution of a huge number of mobile electronics driven by the recent progress observed in the development of the internet of things (IoT), wireless sensor networks (WSN), microelectromechanical systems (MEMS), wearable electronics, implantable medical devices, robotics and artificial intelligence (AI), all of them requiring new compact and efficient sources of energy [2–5]. Foreseen problems related to the limited lifetime and periodic replacement of conventional external power sources, such as batteries, in a large number of devices must be addressed. Fortunately, a continuous reduction in size and power requirements of integrated circuits to values lower than a few µW has been seen last decade [6, 7]. Thus, the concept of nanoenergy was created, meaning the use of nanomaterials and nanotechnology for the creation of integrated power sources able to harvest various forms of energy, such as light, thermal and mechanical [8–12]. Several types of nanogenerators (NG) have thus already been explored, including those for self-powered industrial and health monitoring systems, smart housing and clothing, ambient intelligence, self-powered portable electronic devices, intelligent traffic systems, vibration dampers, wireless power transmitters, etc. [13–15].

Among the various forms of ambient energy, low-level ambient vibrations are ubiquitous, originating from sources such as human motion (walking, talking, breading, typing, etc.), working machinery, vehicles, ocean and lake waves, wind flows, etc., and typically range in acceleration amplitudes of 0.01‒1 g and frequencies of 1‒200 Hz [7, 12, 16, 17]. The four main transduction mechanisms between mechanical and electrical energy are: piezoelectric [8–13, 18–20], electromagnetic [21, 22], electrostatic [23, 24] and triboelectric [25, 26]. In particular, triboelectric nanogenerators (TENGs) have been developed and very widely investigated during the last decade [27–31]. The operation mechanism of TENGs is based on the combined phenomena of triboelectric and electrostatic induction effects [32–35]. The first consists of a contact-induced electrification, where two different material surfaces become electrically charged after physically contacting each other. The second consists of a flow of free charges in an external circuit induced by the electric fields produced by the mechanical separation between the aforementioned surfaces with opposite electrostatic charges. Alternatively, electromagnetic generators (EMGs) have been known since the nineteenth century, being currently widely used in conjunction with various turbines in hydraulic, natural gas, nuclear and coal-based large-scale power generation. The fundamental operation of EMGs is based on the phenomenon of electromagnetic induction, or the generation of an electromotive force in a wire loop by a changing magnetic flux as described by Faraday’s law of induction. Several types of EMGs can be broadly categorized as mechanically into rotatory [36], linear [37] or multidimensional [38] kinetic input and electrically into direct, alternate or variable current output [13, 39–45].

TENGs use a wide range of conventional materials and are currently known to be mechanically flexible, lightweight, cost-effective and easily scalable with low operation frequencies and large bandwidths [46]. EMGs are well-established, efficient, versatile, reliable, effective at large scales, have an easily controllable internal impedance and high frequency of operation. Electrically, TENGs behave as low current sources with high parallel internal impedance because of the electrostatic induction mechanism and the nature of the insulator-to-insulator or insulator-to-metal interface. Mesoscale devices typically have high output open-circuit (OC) voltages (~ 1–1000 V) and low short-circuit (SC) currents (~ 1–1000 µA) and capacitive internal impedance characteristics. Their characteristics of high output voltage and low current and susceptibility to wear, ambient humidity and temperature, as well as low and unstable charge density on tribo-layers, still limit the practical applications of TENGs [46]. EMGs, on the other hand, behave as low voltage sources with low series internal impedance due to the electromagnetic induction mechanism and the high conductivity of the coils. They typically have low output OC voltages (~ 1–1000 mV) and high SC currents (~ 1–1000 mA) and resistive and inductive internal impedance characteristics. As such, both technologies are complementary as explained in the following.

Depending on the environment of operation of self-powered electronic devices, multiple types of energy might be available and the final goal is to power such devices using all available resources. TENG hybrid cells have been developed to simultaneously harvest energy from various sources, such as mechanical [47, 48], solar [49–51], thermal [51] or chemical [49, 52]. Each type of mechanical energy harvesters provides its own benefits and unique advantages/drawbacks [53]. TENG/EMG hybrid cells (E-TENGs) can be used to simultaneously scavenge vibrational energy by taking advantage of their complementarity: high voltage (TENG) and high current (EMG) or, alternatively, to use either of these to meet the requirements of particular applications [54, 55]. Furthermore, they can be used to broaden the operating bandwidth of the nanogenerator due to TENG’s high efficiency at low frequencies and amplitudes of excitation and increased performance of EMGs at high frequencies and amplitudes. In fact, early comparative studies between transduction mechanisms have suggested that piezoelectric and triboelectric energy harvesters provide superior performance in relation to electromagnetic generators at low frequencies and low dimensions [56–60], as well as for small displacement amplitudes of excitation [61]. By taking into account constitutive equations for their respective conversion mechanisms, scaling analysis of the output power of different transducer types as a function of effective material volume (*V*) has shown that it should be roughly proportional to *V*^2^ and *V*^2/3^ for the electromagnetic and electrostatic generators, respectively [8, 56, 58]. Thus, below a critical volume of ~ 0.5 cm^3^ the triboelectric mechanism can become more attractive [58]. Nevertheless, technological difficulties may still be encountered at smaller size scales, e.g., high magnetic flux gradients, assembling components in EMGs and surface potential decay due to imperfect dielectric insulation and stray electric field at the edges in TENGs.

**Fig. 1:**
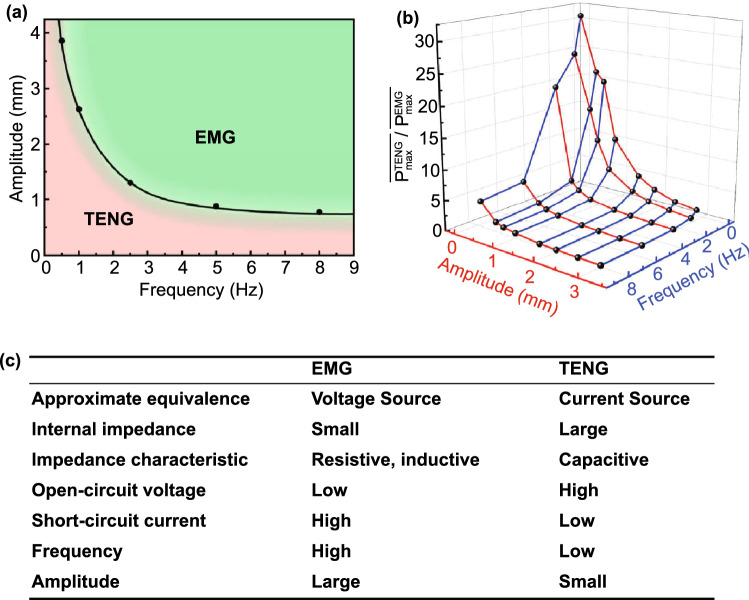
**a** Domain of excitation amplitude and frequency values where the energy harvesting performance of a test EMG or TENG is superior. The light red area denotes the dominant scope of the TENG in low-frequency and small-amplitude while the light green area denotes that of the EMG. **b** Maximum average output power ratio of the EMG and TENG versus amplitude and frequency. **c** Summarized overall comparison table between the complementary characteristics of EMGs and TENGs. Reproduced with permission from Ref. [61]. Copyright 2019, Nano Energy. (Color figure online)

Recently, linear TENGs and EMGs, with similar geometry and size, were fabricated and their electrical output characteristics were systematically studied as a function of the amplitude and frequency of excitation, yielding significantly larger output powers for the TENG in the low-frequency (⪅ 1 Hz) and small-amplitude regime (⪅ 1 mm), as illustrated in Fig. 1a, b [61]. The most important electromechanical characteristics of TENGs and EMGs are summarized in Table 1 and Fig. 1c. Another study compared the applied torque and energy conversion efficiencies between rotational TENGs and EMGs [62]. The input mechanical torque of the EMG was shown to be balanced by the friction and electromagnetic resisting torques, which increased with increasing rotation rate due to Ampère’s force. The input torque of the TENG was balanced by the friction and electrostatic resisting torques, which were nearly constant with the rotation rate. The energy conversion efficiency of the EMG was observed to increase with increasing mechanical power inputs, while the one of the TENG remains nearly constant. These results suggest that the TENG can be superior to the EMG for harvesting mechanical energy with low input powers (⪅ 11.4 mW).

**Table 1:** Summary of the electromechanical parameters of the E-TENGs reported in the literature, generally expressed in terms of maximum peak values: open-circuit voltage (*V*_OC_), short-circuit current (*I*_SC_) and maximum power at a matching load resistance

E-TENG	Triboelectric materials	TENG mode	Energy source	Dimensions	External excitation	Electric characterization TENG	Electric characterization EMG	Electric characterization Hybrid
Rotating segmentally structured disk (Zhang et al.) [63]	Kapton nanorods/Al	Lateral-sliding	General rotation	TENG: 45.2 cm^3^ EMG: 288 cm^3^	20*π* rad/s (600 rpm)	DC $V_{{{\text{OC}}}}$ ≈ 80 V DC $I_{{{\text{SC}}}}$ = 6.96 µA DC Power = 140.4 µW @ 13.8 MΩ	DC $V_{{{\text{OC}}}}$ = 67.6 mV DC $I_{{{\text{SC}}}}$ ≈ 6 mA DC Power = 102.6 µW @ 12.3 Ω	DC $V_{{{\text{OC}}}}$ ≈ 110 mV DC $I_{{{\text{SC}}}}$ ≈ 7 µA DC Power = 209.7 nW @ 18.1 kΩ
Rotating disk (Zhong et al.) [64]	Polyamine/radial-arrayed Cu	Lateral-sliding	General rotation	Φ140 mm × 5 mm	200 rpm	$V_{{{\text{OC}}}}$ = 75 V $I_{{{\text{SC}}}}$ = 330 µA Power = 8.6 mW @ 0.2 MΩ	$V_{{{\text{OC}}}}$ = 0.62 V $I_{{{\text{SC}}}}$ = 57.8 mA Power = 8.4 mW @ 12 Ω	ND
Waterproof rotating disk (Guo et al.) [65]	FEP nanowires/Cu	Sliding free-standing	Wind energy/water flow	Φ100 mm	1600 rpm	$V_{{{\text{OC}}}}$ = 500 V $I_{{{\text{SC}}}}$ = 100 µA Power = 7 mW @ 10 MΩ	$V_{{{\text{OC}}}}$ = 2.9 V $I_{{{\text{SC}}}}$ = 15 mA Power = 4.5 mW @ 60 Ω	$V_{{{\text{OC}}}}$≈ 5 V $I_{{{\text{SC}}}}$ ≈ 2.3 mA Power ≈ 2.9 mW @ ≈ 1.1 kΩ
Rotating-disk-based (Chen et al.) [66]	PTFE/Radial-arrayed Au	Sliding free-standing	General rotation	Φ280 mm	900 rpm	$V_{{{\text{OC}}}}$ = 127 V $I_{{{\text{SC}}}}$ = 4.2 mA Power = 217.8 mW @ 20 kΩ	$V_{{{\text{OC}}}}$ = 33.5 V $I_{{{\text{SC}}}}$ = 39 mA Power = 137.39 mW @ 300 Ω	ND
Rotating-sleeve-based (Cao et al.) [67]	FEP nanowires/Cu	Sliding free-standing	Wind energy	ND	400 rpm	$V_{{{\text{OC}}}}$ = 600 V $I_{{{\text{SC}}}}$ = 150 µA Power = 12.7 mW @ 9 MΩ	$V_{{{\text{OC}}}}$ = 0.85 V $I_{{{\text{SC}}}}$ = 11.5 mA Power ≈ 2.1 mW @ 50 Ω	$V_{{{\text{OC}}}}$ = 48 V $I_{{{\text{SC}}}}$ = 2.2 mA Power ≈ 13 mW @ ≈ 8 kΩ
Rotating-disk-based (Zhang et al.) [68]	PTFE nanostructured/Al	Single-electrode	Wind energy/Road traffic	≈ 3.14 × 10^–4^ m^3^	1000 rpm	$V_{{{\text{OC}}}}$ = 240 V $I_{{{\text{SC}}}}$ = 10 µA Power = 3.4 mW @ 50 MΩ	$V_{{{\text{OC}}}}$ = 7.5 V $I_{{{\text{SC}}}}$ = 9 mA Power = 16.2 mW @ 400 Ω	$V_{{{\text{OC}}}}$ = 3.5 V $I_{{{\text{SC}}}}$ = 5 mA Power = 17.5 mW @ 700 Ω
Ultra-low friction (Wang et al.) [69]	FEP/Cu	Sliding-mode free-standing	Wind energy	≈ Φ65 mm × 40 mm	1000 rpm	$V_{{{\text{OC}}}}$ = 65 V $I_{{{\text{SC}}}}$ = 8.2 µA Power = 438.9 mW/kg @ 40 MΩ	$V_{{{\text{OC}}}}$ = 7 V $I_{{{\text{SC}}}}$ = 100 mA Power = 181 mW/kg @ 80 Ω	ND
Blue energy harvester (Shao et al.) [70]	PVDF nanowires/Al	Contact-separation	Blue energy	ND	100 rpm	$V_{{{\text{OC}}}}$ = 315.8 V $I_{{{\text{SC}}}}$ = 44.6 µA Power_avg_ = 90.7 µW @ ~ 100 MΩ	$V_{{{\text{OC}}}}$ = 0.59 V $I_{{{\text{SC}}}}$ = 1.78 mA Power_avg_ = 79.6 µW @ 318 Ω	ND
Broad-band blue energy nanogenerator (Wen et al.) [71]	FEP nanowires/foam	Sliding-mode free-standing	Blue energy	≈ Φ70 mm × 80 mm	300 rpm, 20 mm	$V_{{{\text{OC}}}}$ = 375 V $I_{{{\text{SC}}}}$ = 14.12 µA Power_avg_ = 15.67 µW/cm^2^ $V_{{{\text{OC}}}}$ = 360 V $I_{{{\text{SC}}}}$ = 8.05 µA Power_avg_ = 4.07 µW/cm^2^	$V_{{{\text{OC}}}}$ = 1.79 V $I_{{{\text{SC}}}}$ = 11.57 mA Power_avg_ = 27.12 µW/cm^2^ $V_{{{\text{OC}}}}$ = 0.51 V $I_{{{\text{SC}}}}$ = 2.91 mA Power_avg_ = 6.33 µW/cm^2^	ND
Easily assembled hybrid (Zhong et al.) [72]	FEP/Cu	Sliding-mode free-standing	Wind energy/water flow	Φ65 mm × 55 mm	500 rpm	$V_{{{\text{OC}}}}$ = 200 V $I_{{{\text{SC}}}}$ ≈ 10 µA Power = 1.05 mW @ 4 MΩ	$V_{{{\text{OC}}}}$ = 5 V $I_{{{\text{SC}}}}$ ≈ 60 mA Power = 58.3 mW @ 20 Ω	ND
Thermomagnetic generator (Ahmed et al.) [73]	FEP/Al	Sliding-mode free-standing	Thermal energy	≈ Φ95 mm × 100 mm	46.5 °C (263 rpm)	$V_{{{\text{OC}}}}$ = 15.6 V $I_{{{\text{SC}}}}$ = 9.82 µA Power = 14.4 µW @ 10 MΩ	$V_{{{\text{OC}}}}$ = 4.56 V $I_{{{\text{SC}}}}$ = 20.61 mA Power = 15.62 mW @ 100 Ω	$V_{{{\text{OC}}}}$ = 15.34 V $I_{{{\text{SC}}}}$ ≈ 20.61 mA Power = 12.1 mW @ 120 Ω
Chaotic pendulum (Chen et al.) [74]	PTFE/Au	Sliding-mode free-standing	Blue energy	Φ100 mm × 167 mm	2.5 Hz	$V_{{{\text{OCpp}}}}$ = 197.03 V $I_{{{\text{SCpp}}}}$ = 3 µA Power = 15.21 µW @ 400 MΩ	$V_{{{\text{OCpp}}}}$ = 1.08 V $I_{{{\text{SCpp}}}}$ = 4 mA Power = 1.23 mW @ 400 Ω	ND
Rotational pendulum (Hou et al.) [75]	FEP/Cu	Contact-separation	Human motion/blue energy	Φ71 mm × 40 mm	10 cm	$V_{{{\text{OC}}}}$ = 230 V $I_{{{\text{SC}}}}$ = 7 µA Power = 0.65 mW @ 10 MΩ	$V_{{{\text{OC}}}}$ = 17.5 V $I_{{{\text{SC}}}}$ = 50 mA Power = 265 mW @ 75 Ω	ND
Soft-contact swing generator (Feng et al.) [76]	FEP/Rabbit hair	Sliding-mode free-standing	Blue energy	≈ Φ100 mm × 50 mm	10 cm, 0.1 Hz	$V_{{{\text{OC}}}}$ = 640 V $I_{{{\text{SC}}}}$ = 4.57 µA Power = 1.29 mW @ 150 MΩ	$V_{{{\text{OC}}}}$ = 2.9 V $I_{{{\text{SC}}}}$ = 11.9 mA Power = 3.5 mW @ 300 Ω	Power = 4.8 mW
Rotating hexagonal prism (Fan et al.) [77]	PDMS pyramids /ITO	Contact-separation	Rotating tire	Side ≈ 50 mm	ND	ND	ND	$V_{{{\text{OC}}}}$ = 40 V $I_{{{\text{SC}}}}$ = 5 mA
Linear magnetic levitation (Hu et al.) [47]	Kapton nanowires/Cu	Lateral-sliding/Contact-separation	General vibration	ND	ND	$V_{{{\text{OC}}}}$≈ 500 V $I_{{{\text{SC}}}}$ ≈ 325 µA Power = 22.5 mW @ 10 MΩ	$V_{{{\text{OC}}}}$≈ 1.4 V $I_{{{\text{SC}}}}$ ≈ 16 mA Power = 5.8 mW @ 90 Ω	$V_{{{\text{OC}}}}$ = 4.6 V $I_{{{\text{SC}}}}$ = 2.2 mA Power ≈ 10 mW @ *R* = 5 kΩ
Elastic-impact-based hybrid (Rahman et al.) [78]	PTFE nanowires/nylon	Contact-separation	Biomechanical energy	ND	1 g	$V_{{{\text{OCpp}}}}$ = 230 V $I_{{{\text{SCpp}}}}$ = 77 µA Power = 1.21 mW @ 9 MΩ	$V_{{{\text{OCpp}}}}$ = 15.10 V $I_{{{\text{SCpp}}}}$ = 115.88 mA Power = 142.42 mW @ 76 Ω	$V_{{{\text{OC}}}}$ = 13.15 V $I_{{{\text{SC}}}}$ ≈ 60 mA Power = 144.1 mW @ 1.5 kΩ
Hybrid energy cell (Wu et al.) [79]	PDMS pyramids/PA	Contact-mode single-electrode	General vibration	45 mm × 45 mm × 200 mm	22 Hz	$V_{{{\text{OCpp}}}}$ = 600 V $I_{{{\text{SC}}}}$ = 3.5 µA Power = 0.25 mW @ 100 MΩ	$V_{{{\text{OC}}}}$ = 3 V $I_{{{\text{SC}}}}$ = 1 mA Power = 0.58 mW @ 10 kΩ	ND
Heaving point absorber harvester (Saadatnia et al.) [80, 81]	PTFE/Nylon	Sliding-mode free-standing	Blue energy	Φ19.05 mm × 203.2 mm	ND	$V_{{{\text{OC}}}}$≈ 100 V $I_{{{\text{SC}}}}$ ≈ 20 µA Power ≈ 115 W/m^3^ @ ≈ 10 MΩ	$V_{{{\text{OC}}}}$≈ 2 V $I_{{{\text{SC}}}}$ ≈ 60 mA Power ≈ 210 W/m^3^ @ ≈ 40 Ω	ND
Traffic generator (Askari et al.) [82]	PTFE/Al	Sliding-mode free-standing	Road traffic	ND	1500 N	$V_{{{\text{OC}}}}$≈ 450 V $I_{{{\text{SC}}}}$ ≈ 150 µA Power ~ 20.92 W/m^3^ @ ≈ 10 GΩ	$V_{{{\text{OC}}}}$≈ 7.5 V $I_{{{\text{SC}}}}$ ≈ 90 mA Power ≈ 50.81 W/m^3^ @ ≈ 100 Ω	ND
Dual Halbach array-based (Salauddin et al.) [83, 84]	PDMS pyramids/Al	Contact-separation	Human motion	65 mm × 26 mm × 18 mm	0.5 g	*V* = 60 V Power = 88 µW @ 10 MΩ	*V* = 1.22 V Power = 11.5 mW @ 32.5 Ω	Power = 3.1 mW @ 700 Ω
E-TENG–PENG (He et al.) [85]	Silicon with carbon nanotubes/ NdFeB	Contact-mode free-standing	General vibration	Φ48 mm × 27 mm	2.5 mm	$V_{{{\text{OC}}}}$ = 15 V $I_{{{\text{SC}}}}$ = 2 µA Power = 78.4 µW @ ≈ 10 MΩ	$V_{{{\text{OC}}}}$ = 7 V $I_{{{\text{SC}}}}$ = 7 mA Power = 38.4 mW @ ≈ 1000 Ω	ND
Integrated E-TENG–PENG (Ma et al.) [86]	PTFE/Cu	Contact-separation	General rotation	115 mm × 100 mm × 30 mm	45 rpm	$V_{{{\text{OC}}}}$ = 250 V $I_{{{\text{SC}}}}$ = 4.1 µA Power = 712.3 µW @ 72 MΩ	$V_{{{\text{OC}}}}$ = 9.8 V $I_{{{\text{SC}}}}$ = 21 mA Power = 30.9 mW @ 200 Ω	ND
E-TENG–PENG (He et al.) [87]	PDMS patterned/Cu	Contact-separation	General vibration	Φ65 mm × 45 mm	0.5 g	$V_{{{\text{OC}}}}$ = 13.3 V Power = 4.6 µW @ ≈ 1.4 MΩ	$V_{{{\text{OC}}}}$ = 362.1 mV Power = 66.5 µW @ ≈ 343.1 Ω	ND
Stacked E-TENG–PENG (Rodrigues et al.) [88]	PTFE/Nylon	Contact-separation	Human walking	50 mm × 30 mm × 23 mm	322.5 N	$V_{{{\text{OC}}}}$≈ 75 V $I_{{{\text{SC}}}}$ = 38 µA Power = 2.9 mW @ ≈ 1 MΩ	$V_{{{\text{OC}}}}$ = 2.9 V $I_{{{\text{SC}}}}$ = 46 mA Power = 33 mW @ ≈ 70 Ω	$V_{{{\text{OC}}}}$ = 75 V $I_{{{\text{SC}}}}$ = 45 mA Power = 32 mW @ ≈ 100 Ω
Linear spring generator (Zhang et al.) [89]	PDMS pyramids/Al	Contact-separation	Human walking	50 mm × 50 mm × 25 mm	ND	$V_{{{\text{OC}}}}$ = 268 V $I_{{{\text{SC}}}}$ = 61 µA Power = 4.9 mW @ 6 MΩ	$V_{{{\text{OC}}}}$ = 4.9 V $I_{{{\text{SC}}}}$ = 3.6 mA Power = 3.5 mW @ 2 kΩ	ND
Linear spring generator (Liu et al.) [90]	FEP/Al	Contact-separation	General vibration	Φ48 mm × 14.8 mm	1 g	$V_{{{\text{OC}}}}$ = 250 V $I_{{{\text{SC}}}}$ ≈ 14 µA Power = 1.09 mW @ 15 MΩ	$V_{{{\text{OC}}}}$ = 0.52 V $I_{{{\text{SC}}}}$ ≈ 0.8 mA Power = 0.28 mW @ 200 Ω	ND
Broadband nonlinear generator (Gupta et al.) [91]	PTFE/ITO	Contact-separation	General vibration	40 mm × 40 mm × 25 mm	2 g	RMS $V_{{{\text{OC}}}}$ = 9.5 V RMS $I_{{{\text{SC}}}}$ = 70 nA RMS Power = 0.166 µW @ 100 MΩ	RMS $V_{{{\text{OC}}}}$ = 55 mV RMS $I_{{{\text{SC}}}}$ ≈ 4 mA RMS Power = 50 µW @ 15 Ω	ND
Linear spring generator (Quan et al.) [92]	PDMS/Al	Contact-separation	General vibration	4625 + 348 mm^3^	ND	$V_{{{\text{OC}}}}$ = 84 V $I_{{{\text{SC}}}}$ = 43 µA Power = 1.2 mW @ 2 MΩ	$V_{{{\text{OC}}}}$ = 9.9 V $I_{{{\text{SC}}}}$ = 7 mA Power = 17.4 mW @ 2 kΩ	ND
Arc-shaped brace structure (Huang et al.) [93]	PTFE/Cu	Contact-separation	General vibration	25 mm × 25 mm × 8 mm	8 m/s^2^ (~ 0.8 g)	$V_{{{\text{OC}}}}$ = 28 V $I_{{{\text{SC}}}}$ = 90 µA Power = 675 µW/cm^2^	$V_{{{\text{OC}}}}$ = 0.1 V $I_{{{\text{SC}}}}$ = 0.8 mA Power = 80 µW/cm^2^	ND
Non-contact nanogenerator (Ren et al.) [94]	Fe_3_O_4_ nanoparticles PVDF/Al	Contact-separation	Biomechanical	55 mm × 30 mm × 20 mm	2 Hz	$V_{{{\text{OC}}}}$ = 88 V $I_{{{\text{SC}}}}$ = 6 µA Power = 0.23 mW @ 25 MΩ	$V_{{{\text{OC}}}}$ = 4 V $I_{{{\text{SC}}}}$ = 10.5 mA Power = 3.4 mW @ 200 Ω	ND
Multifunctional hybrid solar (Shao et al.) [95]	PTFE nanowires/Al	Contact-separation	Blue energy	250 mm × 100 mm × 30 mm	2 Hz	$V_{{{\text{OC}}}}$ = 142 V $I_{{{\text{SC}}}}$ = 23.3 µA Power_avg_ = 31.5 µW @ ~ 100 MΩ	$V_{{{\text{OC}}}}$ = 0.66 V $I_{{{\text{SC}}}}$ = 2.14 mA Power_avg_ = 66.9 µW @ 261 Ω	ND
Honeycomb hybrid (Feng et al.) [96]	PTFE nanowires /Al	Contact-separation/Sliding-mode free-standing	Blue energy	Φ140 mm × 4 mm	3.5 cm, 4 Hz	$V_{{{\text{OC}}}}$ = 550 V $I_{{{\text{SC}}}}$ = 1.25 µA Energy = 21.7 µJ @ 50 MΩ	$V_{{{\text{OC}}}}$ = 3 V $I_{{{\text{SC}}}}$ = 4.65 mA Power_avg_ = 8.23 µW @ 350 Ω	ND
Ship-shaped generator (Wang et al.) [97]	Silicone/Cu	Contact-separation/Sliding-mode free-standing	Blue energy	ND	2 Hz	$V_{{{\text{OC}}}}$ = 290 V $I_{{{\text{SC}}}}$ = 2.8 µA Power = 165 µW @ 20 MΩ	$V_{{{\text{OC}}}}$ = 4.3 V $I_{{{\text{SC}}}}$ = 15 mA Power = 9 mW @ 100 Ω	ND
Sliding linear-grating (Zhang et al.) [98]	Cu/Nylon	Sliding-mode free-standing	Sliding vibration	120 mm × 40 mm × 16 mm	20 m/s^2^ (~ 2 g)	$V_{{{\text{OC}}}}$ = 118.4 V $I_{{{\text{SC}}}}$ = 0.9 mA Power = 102.8 mW @ 0.4 MΩ	$V_{{{\text{OC}}}}$ = 55.7 V $I_{{{\text{SC}}}}$ = 7.7 mA Power = 103.3 mW @ 6 kΩ	ND
Shared-electrode-based (Quan et al.) [99]	FEP/Nylon	Sliding-mode free-standing	Sliding vibration	10 mm × 5 mm	5 m/s^2^ (~ 0.5 g)	$V_{{{\text{OC}}}}$ = 245 V $I_{{{\text{SC}}}}$ = 2.8 µA Power = 0.22 mW @ 200 MΩ	$V_{{{\text{OC}}}}$ = 0.13 V $I_{{{\text{SC}}}}$ = 3.8 mA Power = 0.08 mW @ 40 Ω	ND
Resonant wideband cantilever (Zhu et al.) [100]	PTFE/Cu stopper	Free-standing	General vibration	ND	2 g	$V_{{{\text{OC}}}}$≈ 4.5 V $I_{{{\text{SC}}}}$ ≈ 42 nA Power_avg_ = 30 µW/m^2^ @ ≈ 100 MΩ	Power_avg_ = 38.1 µW/m^2^ @ 40 Ω	ND
Fully enclosed resonant cantilever (Quan et al.) [101]	FEP/Cu	Contact-separation	General vibration	80 mm × 55 mm × 50 mm	ND	$V_{{{\text{OC}}}}$ = 24 V $I_{{{\text{SC}}}}$ = 20 µA Power = 130 µW @ 0.8 MΩ	$V_{{{\text{OC}}}}$ = 0.8 V $I_{{{\text{SC}}}}$ = 0.5 mA Power = 80 µW @ 3 kΩ	ND
Triboelectric-piezoelectric-electromagnetic cantilever (Du et al.) [102]	PTFE nanoparticles/PET	Contact-separation	Rotating tire	35 mm × 40 mm × 57 mm	ND	$I_{{{\text{SC}}}}$ ≈ 0.1 mA Power = 1.2 mW @ 0.4 MΩ	$I_{{{\text{SC}}}}$ ≈ 0.8 mA Power = 7.4 mW @ 40 kΩ	$I_{{{\text{SC}}}}$ ≈ 0.6 mA Power = 8 mW @ 20 kΩ
Air-flow harvester (Wang et al.) [48]	PTFE/Kapton	Contact-separation	Air-flow	67 mm × 45 mm × 20 mm	18 m/s	$I_{{{\text{SC}}}}$ = 55.7 µA Power = 3.5 mW @ 3 MΩ	$V_{{{\text{OC}}}}$ = 3.3 V $I_{{{\text{SC}}}}$ = 2.3 mA Power = 1.8 mW @ 2 kΩ	$I_{{{\text{SC}}}}$ ≈ 4 mA
Air-flow harvester (Wang et al.) [103]	FEP/Cu	Contact-separation	Air-flow	130 mm × 15 mm × 22 mm	18 m/s	$V_{{{\text{OC}}}}$ = 51 V $I_{{{\text{SC}}}}$ = 40 µA Power = 1.7 mW @ 10 MΩ	$V_{{{\text{OC}}}}$ = 3 V $I_{{{\text{SC}}}}$ = 3.7 mA Power = 2.5 mW @ 1 kΩ	ND
Two-dimensional wave harvester (Hao et al.) [104]	Silicone pyramids/Al	Sliding-mode free-standing	Wave energy	100 mm × 88 mm × 43 mm	ND	$V_{{{\text{OC}}}}$ = 75 V $I_{{{\text{SC}}}}$ = 1.2 µA Power = 80 µW @ 100 MΩ	$V_{{{\text{OC}}}}$ = 9 V = 1 mA Power = 14.9 mW @ 1 kΩ	ND
Wrist generator (Quan et al.) [105]	PVB nanowires-PDMS/Nylon	Contact-separation	Human motion	36 mm × 36 mm × 30 mm	ND	$I_{{{\text{SC}}}}$ ≈ 7 µA Power = 100 µW @ 6 MΩ	$I_{{{\text{SC}}}}$ ≈ 4 mA Power = 2.8 mW @ 700 Ω	ND
Water wave energy harvester (Wu et al.) [106]	PTFE/Cu	Sliding-mode free-standing	Wave energy	Φ100 mm	30 rpm	*V*_OC_ ≈ 60 V *I*_SC_ ≈ 1.2 µA	*V*_OC_ ≈ 1.7 V *I*_SC_ ≈ 1 mA	ND
3D full-space nanogenerator (He et al.) [38]	Silicone pyramids/ Polystyrene	Sliding-mode free-standing	General 3D vibration	Φ120 mm	100 mm	*V*_OC_ = 77 V *I*_SC_ = 0.7 µA Power = 18 µW @ 200 MΩ	*V*_OC_ = 2.4 V *I*_SC_ = 1 mA Power = 640 µW @ 1 kΩ	ND
Wrist-wearable generator (Maharjan et al.) [107]	PTFE nanowires/NdFeB	Sliding-mode free-standing	Human walking	Φ13 mm × 120 mm	ND	*V*_OC_ = 19 V *I*_SC_ = 3 µA Power = 0.223 µW/cm^3^ @ 13 MΩ	*V*_OC_ = 8 V *I*_SC_ = 18 mA Power = 5.15 mW/cm^3^ @ 49 Ω	ND
Magneto-mechano-triboelectric generator (Lim et al.) [108]	PFA nanostructured/Al	Contact-separation	AC current/Magnetic field	60 mm × 20 mm	7 Oe	*V*_OCpp_ = 708 V *I*_SC_ = 277 µA Power = 21.8 mW @ 2 MΩ	ND	ND

*ND* Not described

Many designs of hybridized E-TENGs have already been proposed and tested in applications such as general vibration energy harvesting, wheel rotation energy, biomechanical energy, blue energy (wave energy and fluid flow), wind energy, thermal energy, among others. These generators achieved maximum output peak powers up to ca. 100 mW [66, 75, 78], average powers around 1 mW [63, 70, 71] and peak power densities up to 1 mW cm^−3^ [72, 75, 85, 88, 93, 98, 107]. However, no literature reviews are currently available on this topic except of more general ones [109, 110]. Then, this study systematically an deeply analyzes, for the first time, major breakthroughs in emerging hybrid E-TENG technologies, with the emphasis on theoretical transduction mechanisms, structural designs and applications, experimental electromechanical output parameters and future outlook.

## Transduction Mechanism Models

### Transduction Mechanisms of TENGs

Understanding the transduction mechanisms of the nanogenerators is imperative for its rational design. The operation mechanism of TENGs is based on the combined phenomena of triboelectric and electrostatic induction effects [32–35]. Electrostatic induction consists of the redistribution of electric charges induced on an object by the electric force and electric field resulting from the physical separation of triboelectric electrostatic charges of opposite signs as described by the Gauss’s law. The triboelectric effect is due to transfer of electrostatic charges on the surfaces of two different materials in physical contact. The basic mechanism behind this phenomenon is still under active study due to the complex interplay between many factors such as surface topology, air dielectric breakdown, temperature, humidity and contaminants on the surfaces [111–113]. It is generally thought that it is related to the physical process of adhesion between dissimilar molecules during which electrical charges migrate in order to balance the electrochemical potential between the surfaces. The transferred charges can be electrons, ions or material’s species, with the former believed to be of the dominant type in the majority of solid materials [114]. After the two surfaces are separated, some of the extra charges on one type of molecules are kept, resulting in an excess and deficit of electrostatic charge on each surface. These charges can be retained on the surfaces of insulators in the form of electrostatic charges for a long time at room temperature [114]. A quantitative characterization of the triboelectrification at the nanoscale has been demonstrated by an in situ method combining contact-mode atomic force microscopy (AFM) and scanning Kevin probe microscopy (SKPM) [112]. The magnitude of the triboelectric effect can also be quantified by analyzing the energy band structure of the triboelectric pairs [113]. Such model predicts an increase in the triboelectric surface charge density with an increase in the difference between the work functions and average surface densities of states, as well as a decrease in the dielectric thickness [113]. A recent study experimentally measured time-dependent triboelectric surface charge variations at different temperatures based on the thermionic emission effect for different contact-separation tribo-pairs and extracted the corresponding potential barrier heights [115]. The potential barrier height for the metal/polymer pairs was found to always be determined by the polymer type, corroborating the electron transfer as the dominant mechanism, while the results for polymer/polymer pairs indicated possible co-existence of both electron transfer and material/ion transfer mechanisms.

Nearly all pairs of materials exhibit different electron affinities and thus some kind of triboelectric charging [32]. Different materials can be listed in a triboelectric series in order of polarity with materials toward one end acquiring a more positive net charge when contacted with materials toward the other end [116–118]. Materials at the more positive charge end include nylon, glass, poly(methyl methacrylate) (PMMA), aluminum (Al) and copper (Cu), while materials at the more negative end include polytetrafluoroethylene (PTFE), polyvinyl chloride (PVC), polydimethylsiloxane (PDMS), fluorinated ethylene propylene (FEP), Kapton, polyethylene terephthalate (PET) and polyvinylidene difluoride (PVDF) [116, 117]. Most TENGs studied employ combinations of these triboelectric pairs [32]. Beyond the selection of materials with different polarities, further efforts have been made toward the enhancement of the triboelectric charge generation capability of TENGs by controlling the morphology and functionality of the contact surfaces (i.e., nanopatterns and nanoparticles for larger contact areas, friction, permittivity, charge trapping and surface potential differences). Furthermore, various strategies have been implemented toward enhancing the performance of TENGs subjected to a wide range of potentially hazardous environmental conditions such as temperature, humidity, stress, stray electric fields, etc. [32, 119–123].

**Fig. 2:**
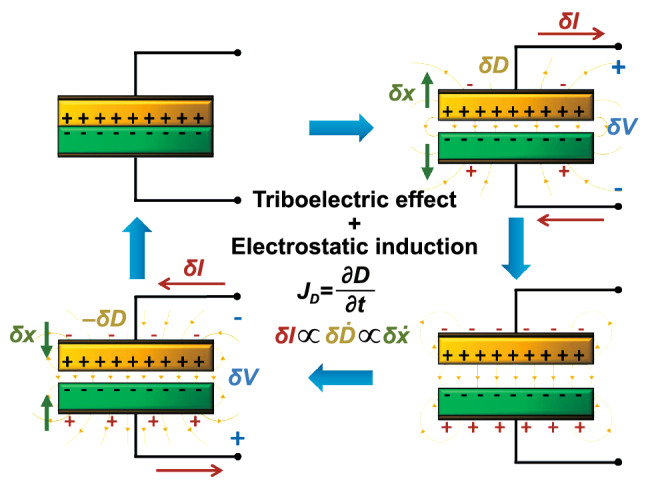
Schematic representation of the working cycle of a contact-separation TENG based on the combined phenomena of triboelectric effect and electrostatic induction. Here, *δx* represents a small change in the distance between the triboelectric layers, *δV* and *δI* a change in voltage and current in an external circuit, respectively, and *δD* a change in electric displacement field. The plus and minus signs in black color represent the electrostatic triboelectric charges on the triboelectric surfaces and in red color the free charges on the electrodes. (Color figure online)

Four fundamental modes of operation of TENGs have been developed, each with unique characteristics and applications including vertical contact-separation, lateral-sliding, single-electrode and free-standing triboelectric layer modes [1, 3, 111]. All of them have in common the presence of, at least, one pair of triboelectric surfaces, where the electrostatic charges are generated, and at least two electrodes, where the free charges accumulate in such a way as to balance the field produced by the electrostatic charge separation. Alternatively, their operation can be understood through an equivalent circuit model where at least two capacitances, formed between the charged surfaces, change with their relative positions, thus inducing a rearrangement of charges. The TENG’s working mechanism is similar to that of electrostatic variable-capacitance generators with the exception that the electrostatic charges are self-generated by the triboelectric effect rather than by an external power source [24]. Figure 2 illustrates an example of the operation cycle of a typical TENG. Here, electrostatic charges of opposite signs initially accumulate on the surfaces of two materials in contact by triboelectrification. A small mechanical separation between the two plates (${\delta} x$) then leads to a separation of electrostatic charges and, consequently, to the buildup of an electric displacement field ($D$) and change in voltage ($\delta V$). Further, displacement current density ($J_{{\text{D}}}$) and flow of free charges through an external circuit ($\delta I$) by electrostatic induction should balance electric field near the conductive electrodes. Subsequently, changes in voltage and current are reversed as the plates are brought back together (Fig. 2). In this manner, mechanical energy, which induces the displacement between plates, is converted into electrical energy flowing into the external circuit. As we will see latter, for infinitesimal displacements the circuit parameters can be shown to be related ca. as: $\delta V + \delta Q/C \propto \delta x$, where $\delta Q$ is the change in free charge and $C$ is the equivalent capacitance of the structure.

**Fig. 3:**
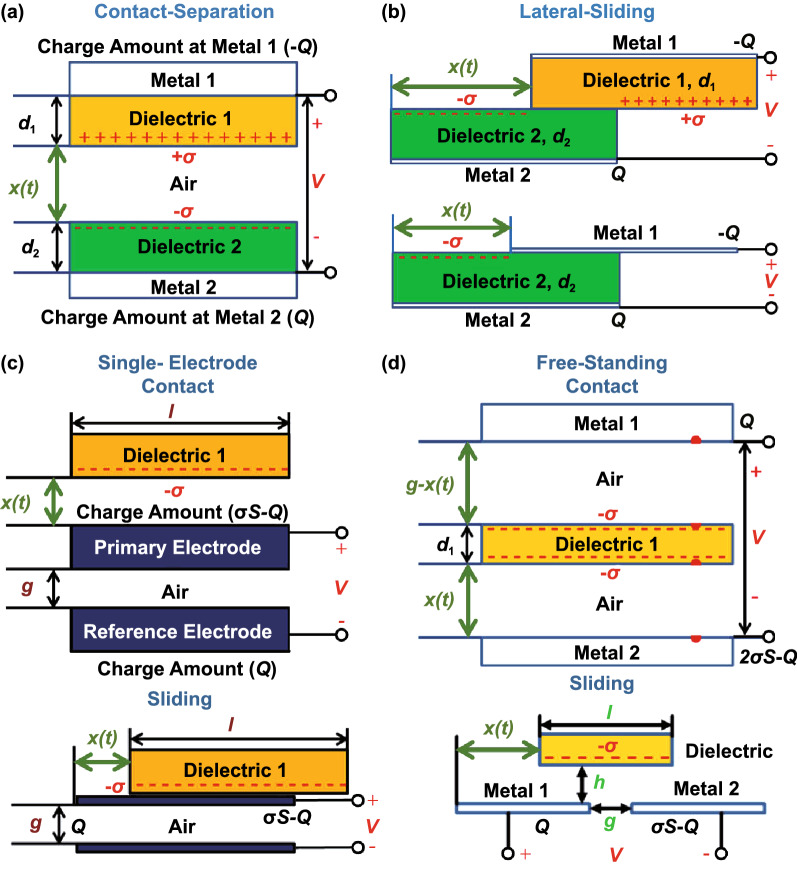
Transduction mechanism models for the four modes of operation of TENGs: **a** Contact-separation [131]. **b** Lateral-sliding (dielectric-dielectric on top and metal-dielectric on bottom) [132]. **c** Single-electrode (contact-mode on top and sliding-mode on bottom) [133]. **d** Contact free-standing mode (contact-mode on top and sliding-mode on bottom) [134]. Mechanically induced time variations of the distance (*t*) between charged triboelectric layers results in a voltage (*V*) build-up and balancing flow of free charges (*Q*) through an external circuit. Adapted from Ref. [1]. (Color figure online)

Models of transduction mechanisms provide valuable understanding and guidance for optimizing the performance of the devices with rational structural design and material selection. The most important equation that describes the electrical output of a TENG is the *V*–*Q*–*x* relationship, which links the voltage between electrodes (*V*), free charge on the electrodes (*Q*) and separation distance between the triboelectric layers (*x*). This relationship can be derived based on classical electrodynamics and continuum mechanics equations (Maxwell’s equations, Lorentz force and balance laws) [124–130]. The schematics of the theoretical transduction models developed for four types of TENGs are shown in Fig. 3.

The vertical contact-separation TENG is the most basic design and is formed by two parallel triboelectric films, which can contact each other, and two conductive electrodes deposited on the top and bottom of the structure [135–137]. After being brought together, the two triboelectric films acquire an opposite surface net charge. When the two surfaces are separated, under an external physical force, an electric potential difference is created. If the electrodes are connected to an external load, free charges will instantly flow between them to balance the electrostatic potential. This mode of operation is well known for its simple design and fabrication, great robustness and high output power density [1]. It is thus adequate for intense cyclic motions and intermittent impacts. On the downside, their use requires a cavity with changing volume, which can introduce unit packaging problems.

**Fig. 4:**
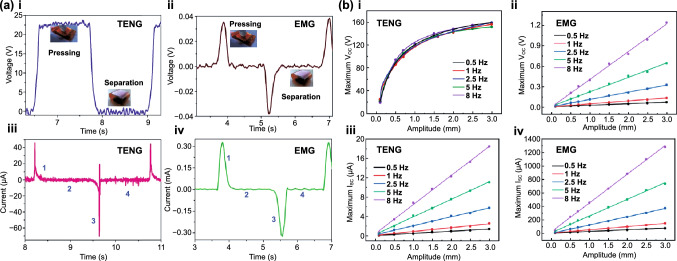
**a** Typical time response of an hybrid generator with a coupled linear EMG and contact-separation TENG to a square-wave mechanical finger tap input: (i) OC voltage of the TENG; (ii) OC voltage of the EMG; (iii) SC current of the TENG; (iv) SC current of the EMG. The voltage of the TENG is ca. proportional to the displacement, while the current of the TENG, voltage and current of the EMG are ca. proportional to the velocity. Reproduced with permission from Ref. [93]. Copyright 2017, Creative Commons Attribution 3.0 Unported Licence. **b** Typical variation of the output OC voltage amplitude and SC current amplitude of an EMG and TENG with the amplitude of displacement and frequency of an excitation sinusoidal input. (i) OC voltage of the TENG; (ii) OC voltage of the EMG; (iii) SC current of the TENG; (iv) SC current of the EMG. The voltage from the EMG increases linearly with the amplitude, while the voltage from the TENG increases linearly up to a saturation value. The current from both generators increases linearly with the amplitude. The voltage from the EMG increases linearly with the frequency; differently, the voltage from the TENG follows an increasing trend up to a stationary value. The current from both generators increases linearly with the frequency. Reproduced with permission from Ref. [61]. Copyright 2019, Nano Energy. (Color figure online)

The scheme of a simple transduction model for the contact-separation mode with two dielectric triboelectric layers is shown in Fig. 3a [131, 138]. The two-dimensional model considers two uniform dielectric parallel layers on top and bottom with surface area $S$, thickness $d_{1}$ and $d_{2}$ and relative dielectric permittivity $\varepsilon_{{{\text{r}}1}}$ and $\varepsilon_{{{\text{r}}2}}$, respectively, and separated vertically by a distance $x$. When contacting each other, the two triboelectric layers are assumed to acquire uniform static surface charge densities $\sigma$ of opposite signs that do not decay with time. Since the separation between charges is negligible in this scenario, the net charge is null and there is no electric field generated in the medium. As an external physical force increases the distance $x$ between the dielectrics, the static charges are separated and a potential difference ($V$) is generated between the electrodes. This voltage then drives a current ($I$) to flow through an external load building up opposite net free charges ($Q$) on the electrodes. This current is equal to the time derivative of the free charge ($I = \dot{Q}$). For given surface charge densities $\sigma$ and $Q/S$ and at any given instant, the electric potential and field in each region between the plates can be calculated using the Poisson’s equation for electrostatics. When the thickness of the whole structure is small compared to its length and width (i.e., for sufficiently small $x$), the plates behave approximately like infinite planes with constant surface charge density and the electric fields have only vertical components that are easy to obtain analytically from the integral form of Gauss’s law (i.e., $E_{z} \left( z \right) = \sum\nolimits_{i} {\left( {\sigma_{i} /2\varepsilon_{{\text{r}}} \varepsilon_{0} } \right)\left( {z - z_{i} } \right)/\left| {z - z_{i} } \right|}$, with $\sigma_{i}$ and $z_{i}$ being the surface charge density and position of the *i*th plane, respectively). Thus, the $V$–$Q$–$x$ relationship for contact-separation TENG in this case is given as follows (with $V = - \smallint E_{z} \left( z \right){\text{d}}z$) [131]:

1 $$V = - \frac{1}{{\varepsilon_{0} }}\frac{Q}{S}\left( {\frac{{d_{1} }}{{\varepsilon_{{{\text{r}}1}} }} + \frac{{d_{1} }}{{\varepsilon_{{{\text{r}}1}} }} + x} \right) + \frac{1}{{\varepsilon_{0} }}\sigma x$$

where $\varepsilon_{0}$ is the permittivity of vacuum. This equation shows that the voltage changes proportionally to the displacement $x$, while the current ($I$ = $\dot{Q}$) varies ca. proportionally with the velocity of separation between the plates ($\dot{x}$). Thus, the time response of a contact-separation TENG to a square-wave variation of $x$ (i.e., pressing/releasing cycles) is shown in Fig. 4a (i, iii), with the OC voltage taking ca. the same form as the square-wave input and the SC current being ca. proportional to its derivative due to the capacitive nature of the system. Furthermore, in the general case for a sinusoidal time changing displacement input with a constant amplitude and changing frequency, the trend of the TENGs output voltage amplitude is to remain constant, while the current amplitude increases linearly with the frequency (Fig. 4b (i, iii)) [61, 93]. In practice, the output voltage only grows with the displacement amplitude $x$ up to a saturation point due to the non-infinitely large area of the plates and the resulting edge effect (Fig. 4b (i)) [61]. The OC voltage ($V_{{{\text{OC}}}}$), SC charge ($Q_{{{\text{SC}}}}$) and effective capacitance ($C = Q_{{{\text{SC}}}} /V_{{{\text{OC}}}}$) can be derived from Eq. (1) by setting $Q$ = constant and $V$ = 0, respectively. This approximate solution also shows that a small change in the distance between plates ($\delta x$) produces a change in the OC voltage ($\delta V_{{{\text{OC}}}}$), which increases linearly with $\delta x$ and $\sigma$ and a change in the SC charge ($\delta Q_{{{\text{SC}}}}$), which, in turn, increases ca. linearly with $\delta x$, $\sigma$ and $S$, and is maximal when $x$ is close to zero and the effective thickness of the dielectrics ($d_{0} = d_{1} /\varepsilon_{{{\text{r}}1}} + d_{2} /\varepsilon_{{{\text{r}}2}}$) is small. Consequently, the maximum harvestable average power should also increase with these parameters (i.e., as $\delta V_{{{\text{OC}}}} \cdot \delta \dot{Q}_{{{\text{SC}}}} \approx \propto \sigma^{2} S/d_{0}$) and this suggests that structures with high triboelectric charges $\sigma$, large areas $S$ and small dielectric thicknesses $d_{0}$, are desirable. The triboelectric surface charge density is thus the most important material parameter to be quantified that defines the performance of such devices [117].

Since the power delivered by a single contact-separation TENG is still too low for high-power consumption electronics, multi-layered TENGs have been investigated [122]. Structures with a metal electrode (noted A) and dielectric layer (noted B) can be arranged in a symmetrical (ABBA) or alternate (ABAB) pattern. The proposed model revealed a larger inherent capacitance due to the parasitic capacitance effect between units in the alternate layered TENG [122]. The symmetrical structure thus yields higher OC voltage with less transferred charges and higher output peak power. When decreasing the size of the layered TENG, the influence of the electric field edge effect was also shown to effectively enhance the air capacitance and thus the parasitic capacitance and output current for the alternate TENG.

The lateral-sliding-mode TENG, like the contact-separation TENG, is composed by two triboelectric layers and two top and bottom electrodes [139, 140]. However, this mode is designed to operate with a relative in-plane lateral-sliding between the triboelectric layers. The sliding motion creates opposite charges and a lateral displacement between the charged plates, resulting in a separation of electrostatic charge and, thus, a potential difference, which drives free charges through an external load. This mode offers a more effective generation of triboelectric charge since: (i) the thorough contact associated with the sliding motion [139]; (ii) a grating structure can be used to enhance the separation of electrostatic charges for smaller applied displacements [140–142], although it might also suffer from intensive wear. Thus, it is useful for effective harvesting energy from planar motions, disk rotations and cylindrical rotations.

The basis of the transduction model of the lateral-sliding-mode TENG is illustrated in Fig. 3b [132, 143]. The design here is equivalent to the contact-separation mode architecture, except that a length directed lateral separation between the plates with magnitude $x$ is considered instead. When the displacement $x$ is non-null, there is a non-overlapping net density of electrostatic surface charge $\sigma$, with different sign, on each triboelectric surfaces in no-contact. This distribution of charges thus produces an electric field in the medium with components parallel and perpendicular to the displacement and consequent built-up of free charges ($Q$) on the electrodes. In the general case, an analytic solution to the equations cannot be found, so numerical methods, such as finite element method (FEM), must be used to find the distribution of potential and free charge [132]. If the vertical distance between layers is small compared to their width ($w$) and length ($l$), the edge effects can be neglected and most of the electric field will point in the vertical direction pulling a certain amount of opposite free charges to the overlapping and non-overlapping regions of the electrodes. Under SC conditions, the non-overlapped region of the electrodes will contain ca. the same amount of charge as in the triboelectric layer, i.e., $\sigma wx$, uniformly distributed. Under OC conditions, the overlapped region should also contain ca. this same amount of charge with opposite sign to cancel the net charge on the electrode. Using both the Gauss’s law and gradient theorem in the overlapped region to calculate the voltage drop, the linear $V$–$Q$–$x$ relationship for the lateral-sliding-mode TENG is given as follows (valid for displacements $x$ and thicknesses $d_{0}$ significantly smaller than the length $l$) [132]:

2 $$V = \frac{1}{{\varepsilon_{0} }}\left( {\frac{{d_{1} }}{{\varepsilon_{{{\text{r}}1}} }} + \frac{{d_{1} }}{{\varepsilon_{{{\text{r}}1}} }}} \right)\frac{1}{l - x}\left( { - \frac{Q}{w} + \sigma x} \right).$$

As in the case of the contact-separation mode, Eq. (2) shows that the voltage changes ca. proportionally with the displacement $x$, for small values of $x$, while the current varies ca. proportionally with the velocity of the displacement ($\dot{x}$), although in this case $x$ is further allowed to take on negative values. Thus, for harmonic inputs, the output OC voltage tends to remain constant, while the SC current increases linearly with the frequency [141]. A small change in the displacement ($\delta x$) can also be shown to induce a change in the OC voltage ($\delta V_{{{\text{OC}}}}$), which increases linearly both with $\sigma$ and the effective thicknesses of the dielectrics ($d_{0}$), and it is maximal when $x$ is close to $l$ and $l$ is small. The change in the SC charge ($\delta Q_{{{\text{SC}}}}$), on the other hand, increases linearly with $\sigma$ and $w$. The maximum harvestable average power also increases with these parameters (i.e., as $\delta V_{{{\text{OC}}}} \cdot \delta \dot{Q}_{{{\text{SC}}}} \approx \propto \sigma^{2} wd_{0} /l$) and, thus, structures are optimized with a large triboelectric charge generation and width-to-length ratio (up to a certain point at which the fringe field effects start to become noticeable). A grating structure, with multiple lateral-sliding units side-by-side, is ideal in this case because it permits the induction of a larger net non-overlapped charge for smaller displacements, thus increasing the amount of charge $Q$ in the electrodes and reducing the effective length $l$ in the previous expressions [144].

The single-electrode mode TENG was designed to harvest energy from the relative motion between a freely moving object and the device itself through electrostatic induction [133, 145]. Here, one of the triboelectric layers is external to the device and has no electrical connection, while the other is part of one or two electrodes connected to a load circuit. The relative sliding or separation motion between the layers results in the potential drop accompanied by a flow of balancing free charge through the load. This configuration exhibits an electrostatic screening effect between parallel conductive layers [133], but it can be used in applications where one of the triboelectric layers is free to move and independent from the device body, such has a rotating tire [146].

Single-electrode TENGs can be operated in a contact-mode or sliding-mode. Figure 3c depicts an example of a transduction model for the case of a conductor-to-dielectric single-electrode TENG operating in a contact mode [133, 138, 147]. The triboelectric pair is composed of a free dielectric layer and a conductive primary electrode with thickness $d_{1}$ and $d_{m}$, respectively. The distance between these two layers is $x$ and can be changed with time. A second reference electrode is placed a distance $g$ below the primary electrode. With $\sigma S$ representing the net charge induced in the dielectric and primary electrode by the triboelectric effect, and $Q$ the net charged transferred from the primary to the secondary electrode, the total amount of charge in the primary electrode is estimated to match $\sigma S - Q$, because of charge conservation. In general, a numerical FEM method is required to compute the electrical potential, although the structure can be modeled as a three-capacitance equivalent circuit having a $V$–$Q$–$x$ relationship [133]:

3 $$V = \sigma S\frac{{C_{2} }}{{C_{1} C_{2} + C_{2} C_{3} + C_{3} C_{1} }}\left( {1 - \frac{Q}{\sigma S}\left( {1 + \frac{{C_{1} }}{{C_{2} }}} \right)} \right)$$

where $C_{1}$, $C_{2}$ and $C_{3}$ are capacitances between the electrical nodes dependent on the dimensions and distances between the layers. In general, the OC voltage ($V_{{{\text{OC}}}}$) and SC charge ($Q_{{{\text{SC}}}}$) increase with $x$ up to a saturation value. $V_{{{\text{OC}}}}$ increases and $Q_{{{\text{SC}}}}$ decreases with the electrode gap distance ($g$), so that, for a given optimal value, the maximum average power is obtained. $Q_{{{\text{SC}}}}$ increases with the area of the surfaces ($S$), while $V_{{{\text{OC}}}}$ increases up to an optimal value and, then, starts decreasing. Thus, there is also an optimal value of $S$ for maximum output power. Therefore, unlike the case of paired electrode TENGs, single-electrode TENGs are not optimized for direct scale-up processing: the best strategy is to use the parallel electrical connection of multiple spaced units [133].

Similarly to the single-electrode TENG, the free-standing mode TENG contains an electrically disconnected free-moving triboelectric layer, as well as a pair of symmetric plates comprising electrodes placed above and below the former, or side-by-side, connected electrically between themselves and to an external load [148, 149]. The free layer, once charged via a triboelectric process, moving about the two plates, creates an asymmetric charge distribution and consequent flow of charge through the load. This mode does not exhibits electrostatic screening, thus having a large charge transfer efficiency. Since it does not necessarily require a direct contact between the surfaces, the wear can be minimized [149]. The free-standing TENGs can, thus, be used to harvest energy from arbitrary moving objects such as vehicles [150] or human motion [148].

Free-standing TENGs can be operated in a contact-mode or sliding-mode. An example of a transduction model developed for the case of a dielectric free-standing TENG operating in a contact-mode is illustrated in Fig. 3d [134, 147]. The free-standing layer consists of a dielectric with thickness $d_{1}$ moving between two electrodes separated by an air gap with length $g$. During operation, the free-standing layer moves in the vertical direction (the distance between its lower surface and the bottom electrode given by $x$). From the triboelectric effect, the top and bottom surfaces of the dielectric acquire a net charge ${-}\sigma S$, while one of the electrodes is estimated to have a net charge $Q$ and the other $2\sigma S - Q$, by the charge conservation. Using the concept of nodes, and an equivalent circuit with three capacitors or an infinite charged plane model, the $V$–$Q$–$x$ relationship for the free-standing mode TENG can be given by [134]:

4 $$V = - \frac{1}{{\varepsilon_{0} }}2\sigma x + \frac{{d_{1} /\varepsilon_{{{\text{r}}1}} + g}}{{\varepsilon_{0} }}\frac{Q}{S} - \frac{1}{{\varepsilon_{0} }}\frac{{d_{1} }}{{\varepsilon_{{{\text{r}}1}} }}\sigma .$$

This equation shows that, unlike in the contact-separation TENG described by Eq. (1), both the OC voltage and SC charge change linearly with the $x$ distance with no saturation trend (although the maximum value of $x$ is limited by $g$). The maximum harvestable power varies similarly with the contact-separation case (i.e., as $\delta V_{{{\text{OC}}}} \cdot \delta \dot{Q}_{{{\text{SC}}}} \approx \propto \sigma^{2} S/\left( {d_{0} + g} \right)$). Structures with large triboelectric charge generation, areas and small dielectric thicknesses should thus be optimal. A model for the sliding-mode of a free-standing TENG has also already been developed. Its greatest advantage in relation to the lateral-sliding structure should be the superior tolerance to the free-standing height ($h$), ensuring high performance in the non-contact mode [134].

**Fig. 5:**
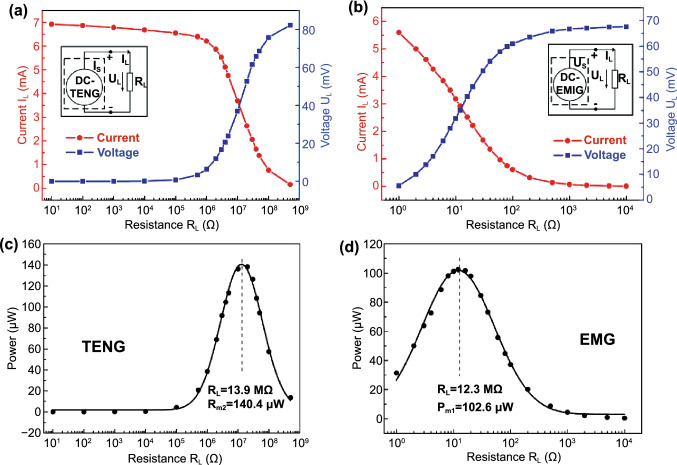
Typical dependence of the harmonic output voltage amplitude, current amplitude and average power of a TENG and EMG with the value of an external load resistance and Norton equivalent circuits. **a** TENG voltage and current; **b** EMG voltage and current. **c** TENG power; **d** EMG power. As depicted, the TENG yields high output voltage and low current, while the EMG yields high output current and low voltage. Reproduced with permission from Ref. [63]. Copyright 2014, Advanced Materials. (Color figure online)

The first-order lumped parameter equivalent circuit model of a general TENG is a current source, with output $I_{{{\text{SC}}}}$, in parallel with a capacitor, with capacitance $C$ or equivalently a voltage source, with output $V_{{{\text{OC}}}}$, in series with the capacitor, which are consequences of the Norton’s and Thévenin’s theorems, respectively [124]. TENGs are usually connected to a resistive load ($R$). The voltage in the $V$–$Q$–$x$ relationships can, thus, be combined with Ohm’s law ($V = RI$), resulting in a first-order ordinary differential equation (ODE) with dependent variable $Q$ written as a function of time and with $x$ as the input displacement term, which is itself a function of the external applied forces. This equation can then be analytically solved with given initial conditions. The peak values of the output voltage, current and power measured for different loads are shown to follow a characteristic curve that can be divided into three working regions, as shown in Fig. 5 [124]. For low values of the load, the circuit features a small resistance to the transference rate of free charge. Thus, the value of $Q$ always stays close to its maximum value under SC conditions ($Q_{{{\text{SC}}}}$), almost completely screening the electric field from the separated triboelectric charges, so that $V$ is ca. null and $I$ is maximized. As the resistance increases, the transference rate of free charge diminishes and, consequently, the peak voltage increases and the peak current decreases. Since the instantaneous power on the load is equal to the voltage times the current, it attains a maximum peak value in this region. For the case of an harmonic time changing displacement $x$ with angular frequency $\omega$, and in the linear circuit approximation ($V = - Q/C + V_{OC}$), the output average power on the load ($P = VI = \mathop \smallint \limits_{0}^{T} VI{\text{d}}t/T$) can be shown to take a maximum value of $P_{\max } = \left( {1/4} \right)V_{{{\text{OC}}}} I_{{{\text{SC}}}}$ for impedance matching conditions when the resistance equals $R = 1/\omega C = V_{{{\text{OC}}}} /I_{{{\text{SC}}}}$. More generally, for a linear circuit with a complex internal impedance $Z$, the output average power should be of the form $P = \left( {1/2} \right)RII^{*}$, taking a maximum value $P_{\max } = \left( {1/4} \right)V_{{{\text{OC}}}} I_{{{\text{SC}}}} \left( {\left| Z \right|/\left[ {\left| Z \right| + Z^{\prime } } \right]} \right)$ for an optimal resistance $R = \left| Z \right| = V_{{{\text{OC}}}} /I_{{{\text{SC}}}}$, (the asterisk indicates the complex conjugate and the apostrophe the real part). For large values of the load, the flow of charges is very slow and, thus, there is very low screening of the triboelectric charges and $V$ approaches its maximum OC value ($V_{{{\text{OC}}}}$) while $I$ is ca. null. This kind of behavior with three working regions is common for all types of TENGs [124]. The resistive output and charging characteristics of TENGs are critically important for the optimization of its energy harvesting capabilities.

### Transduction Mechanisms of EMGs

EMGs are the type of electrical generators currently most used, including in conjunction with turbines in commercial large-scale power plants based on fossil fuels, nuclear, hydrodynamic and wind power [13, 39–45]. The fundamental operation of EMGs is based on the phenomenon of electromagnetic induction or the generation of an electromotive force in a wire loop by a changing magnetic flux, as described by the Faraday’s law of induction. Usually, EMGs consist of a moving part and a fixed part comprising coils and/or permanent magnets. Permanent magnets or coils driven by a current are used to generate static or time changing magnetic fields. Electromotive forces are, then, induced in a coil by the time changing magnetic fields or by its movement or deformation in relation to the magnetic field lines. According to the Lenz’s law, the generated electric currents produce magnetic fields opposing the change of the magnetic flux, thus producing a force opposing its relative motion (i.e., damping) by the conservation of momentum. Therefore, the generators convert part of the kinetic energy of its moving parts into electrical energy in the form of currents and voltages in an external circuit.

**Fig. 6:**
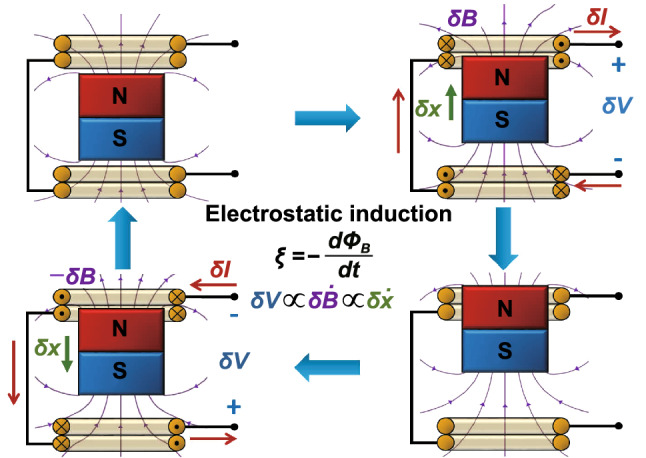
Schematic representation of the working cycle of a linear magnet/coil EMG based on the phenomenon of electromagnetic induction. Here, *δx* represents a small displacement of the permanent magnet in relation to the coils, *δV* and *δI* a change in voltage and current in an external circuit, respectively, and *δB* a change in magnetic induction field. The dots and crosses in the loops of the coils represent currents flowing in the direction out of the plane and into the plane, respectively. (Color figure online)

Several types of EMGs can be broadly categorized: mechanically into rotatory [36], linear [37] or multidimensional kinetic input [38], and electrically into direct or alternate current [13, 39–45]. Figure 6 shows an example of the operation cycle of a linear EMG. Here, a small displacement of a permanent magnet in relation to a pair of coils ($\delta x$) results in a change in magnetic flux ($\Phi_{B}$) flowing through the coils and, consequently, the generation of an electromotive force ($\xi$) and voltage change ($\delta V$) by electromagnetic induction, together with a flow of free charges through an external circuit ($\delta I$). Subsequently, as the magnet moves in the opposite direction, the changes in voltage and current are reversed. For infinitesimal displacements and low frequencies of excitation, the circuit parameters can be shown to change ca. as: $\delta V + R_{I} \delta I \propto \delta \dot{x}$, where $R_{I}$ is the equivalent resistance of the coils.

As in the case of TENGs, the relationship between the output voltage ($V$), current ($I$) and dynamic parameters of the system, provides a very important insight into its behavior and performance. This relationship can be derived using classical electrodynamics and rigid body dynamics, most generally described by the Maxwell’s equations, Lorentz force and balance laws for mass, momentum and energy. General linear kinetic spring-mass models for resonant generators, comprising a mass ($m$), a spring and a damper, with mechanical or parasitic ($\zeta_{{\text{M}}}$) and electromechanical or extraction ($\zeta_{{\text{E}}}$) dimensionless damping ratios, reveal a very large enhancement of the steady state displacement amplitude and energy extraction when the excitation frequency matches the natural resonance frequency of the systems [13, 16, 151, 152]. For a sinusoidal time changing input acceleration with amplitude $A$ and angular frequency $\omega$, the amplitude of the steady state displacement phasor of the mass takes the following form [13, 16, 151, 152]:

5 $$\left| {\hat{x}} \right| = \frac{A}{{\sqrt {\left( {\omega_{0}^{2} - \omega^{2} } \right)^{2} + \left( {\omega \omega_{0} 2\zeta } \right)^{2} } }}$$

where $\omega_{0}$ is the natural resonance angular frequency of the system and $\zeta$ is the total damping ratio, which is equal to the sum of the mechanical $\zeta_{{\text{M}}}$ and total electromechanical $\zeta_{{\text{E}}}$ (due to internal and external loads) damping ratios. The value of the resonance frequency of the system can be tuned to the frequency of an excitation source by controlling its geometry, effective stiffness and mass density. The average output electrical power extracted by such systems operating under resonance conditions can be computed as follows [13, 16, 151, 152]:

6 $$\left\langle P \right\rangle_{{{\text{Res}}}} = \frac{1}{4}\frac{{mA^{2} }}{{\omega_{0} }}\frac{{\zeta_{{\text{E}}} }}{{\left( {\zeta_{{\text{M}}} + \zeta_{{\text{E}}} } \right)^{2} }}.$$

Therefore, for small enough amplitudes of excitation, the electrical output power should be maximized when both damping factors are matched, which can be achieved by controlling the electromechanical damping ratio through the load impedance of the circuit. Furthermore, we should note that higher damping ratios are also associated with larger bandwidths of operation of the generators [10].

**Fig. 7:**
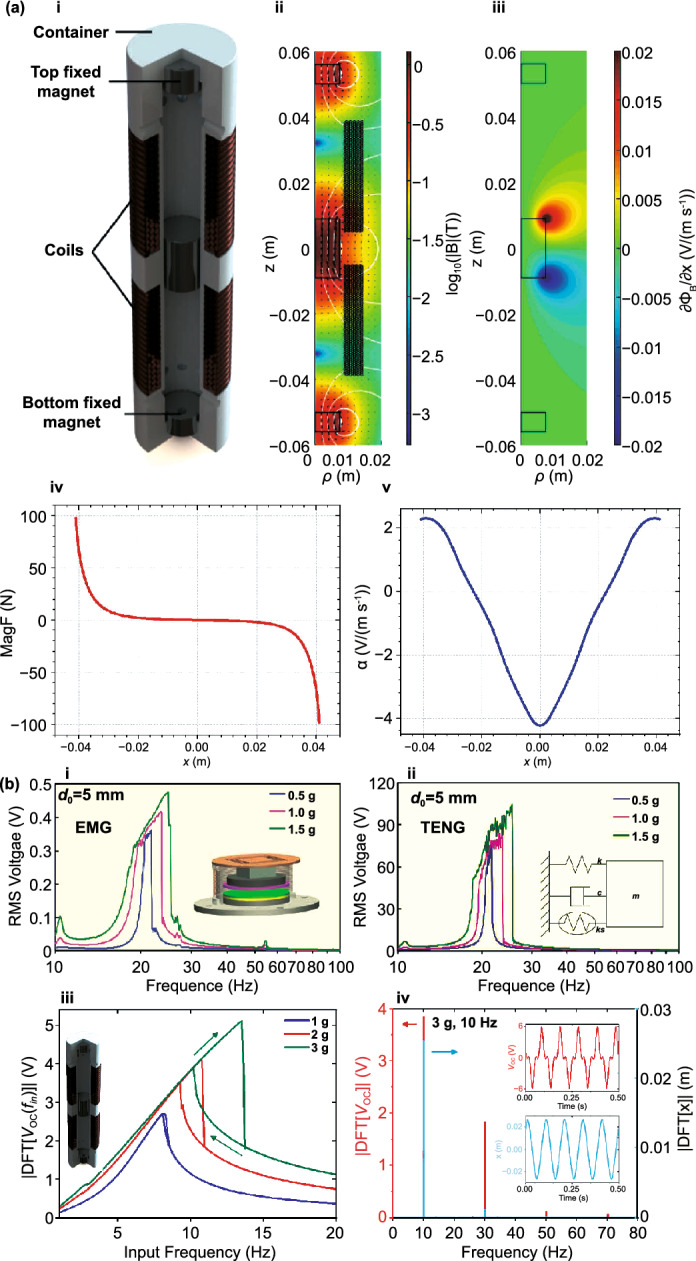
**a** Schematic representation of a cylindrical resonant magnetic levitation EMG. (i) Section view of the levitating system composed of a central free levitating magnet, two fixed magnets with a different magnetization direction and two wound up coils. Reproduced with permission from Ref. [153]. Copyright 2020, Applied Energy. (ii) Magnetic induction field (*B*) lines (in white) and direction arrows (in black) and corresponding absolute value in color scale. (iii) Variation of the magnetic flux (*ΦB*) through a loop of wire with the variation of the position of the free-magnet (*x*), equivalent to the electromechanical coupling factor (*α*) for a single loop. (iv) Total magnetic restoring force (Mag) exerted on the free-magnet *vs* position of the magnet calculated using Bessel functions. (v) Electromechanical coupling coefficient (*α*) for the two coils connected in series versus position of the magnet calculated using elliptic integrals. **b** Experimental frequency response of a nonlinear impact resonant hybrid generator with a coupled (i) magnet–spring–coil EMG and (ii) contact-separation TENG to a harmonic input with acceleration amplitudes of 0.5 g, 1.0 g and 1.5 g. The frequency here was swept from lower to higher values, showing a broad hardening resonant peak, at higher accelerations, of the OC RMS voltage with two critical points at ca. 19 Hz and 25 Hz similarly to a Duffing oscillator’s response. For the lower acceleration of 0.5 g, the system behaves ca. as in the linear regime characterized by a simple sharp resonant peak at ca. 21 Hz. Adapted from Ref. [90]. (iii) Absolute value of the discrete Fourier transform (DFT) of the OC voltage (at the same frequency as the input) response of the cylindrical nonlinear magnetic levitation EMG to a harmonic input with acceleration amplitudes of 1 g, 2 g and 3 g, and with increasing and decreasing frequency numerically calculated. (iv) Absolute values of the discrete Fourier transforms of the OC voltage (*V*_OC_) and displacement (*x*) as a function of the output frequency for the same system, with an input excitation of 3 g and 10 Hz, and corresponding time responses shown in the insets. (Color figure online)

One important class of resonant EMGs are the linear magnetic levitation systems, as represented in Fig. 7a (i) [153]. Their basic architecture consists of a hollow container inside of which are placed at least three permanent magnets, each one with alternate polarity to repel its closer neighbors, and surrounded by one or more coils. Several models have been developed to describe this type of systems [153]. The levitating magnet, assumed to move only in one dimension (described by position $x$ at a time $t$), is subjected to nonlinear magnetic restoring forces ($^{{{\text{Mag}}}} F\left( x \right)$) due to gradients of the magnetic fields produced by the fixed magnets, as depicted in Fig. 7a (ii). This force is highly nonlinear with the position of the free-magnet (Fig. 7a (iv)) and allows one to tune the angular natural frequency of the system ($\omega_{0} \left( x \right) = \sqrt {k/m}$, $k = - \partial^{{{\text{Mag}}}} F\left( x \right)/\partial x$), e.g., by controlling the distance between fixed magnets [154]. A current flowing through the coil also produces a Lorentz force on the levitating magnet ($^{Lrz} F\left( {x,I} \right)$) proportional to the current amplitude [155–157]. In operation, external forces applied to the container compels it to move in space and, in the non-inertial frame of reference of the container, the levitating magnet appears to be subjected to resulting inertial forces [155, 158, 159]. This forcing term ($^{{{\text{Forcing}}}} F\left( {x,t} \right) = m\left[ { - \sin \left( \theta \right)\sin \left( \varphi \right)\ddot{T}_{1} + \sin \left( \theta \right)\cos \left( \varphi \right)\ddot{T}_{2} - \cos \left( \theta \right)\ddot{T}_{3} + x\left( {\dot{\theta }^{2} + \sin^{2} \left( \theta \right)\dot{\varphi }^{2} } \right)} \right]$, in terms of a translation vector ${\varvec{T}}$ and intrinsic $z - x^{\prime } - z^{\prime \prime }$ Euler angles *φ, θ, ψ* describing the container position in space at a given time) perturbs the system from its position of equilibrium usually leading to oscillations of the levitating magnet around this point with the natural frequency of the system. The position change of the magnet in relation to the coils leads to changes in magnetic flux ($\Phi_{B}$) flowing through them and, thus, the induction of an electromotive force ($\xi$) proportional to its velocity and quantified by Faraday’s law ($\xi = - {\text{d}}\Phi_{B} /{\text{d}}t$). The derivative of the total flux flowing through the coils, in relation to the position of the levitating magnet (or its additive inverse), is the so-called electromechanical coupling coefficient of the system ($\alpha \left( x \right) = \partial \Phi_{B} /\partial x = - \xi /\dot{x}$) which relates the output voltage with the velocity of the magnet [154, 158, 160]. This coefficient quantifies the coupling between the mechanical and electromagnetic sub-systems and takes a maximum absolute value (with different signs) when the top or bottom edge of the levitating magnet is at the center position of a loop of wire, as shown in Fig. 7a (iii) and in general increases with the number of loops in the coil [37, 154]. For cylindrical containers, the electromechanical coupling factor changes with the position of the free-magnet, as shown in Fig. 7a (v), taking a maximum absolute value when the magnet has its two vertices in the interior region of two different coils followed by a null value when the magnet is completely inside a single coil. A second local maximum also occurs when only one of the vertices is inside a single coil. The dynamics and electrodynamics of the system result in a time-dependent second-order nonlinear system of ODEs [153, 155, 161]:

7 $$\left\{ {\begin{array}{*{20}l} {m\ddot{x} - \;^{Damp} F\left( {\dot{x}} \right) - \;^{Lrz} F\left( {x,I} \right) - \;^{{{\text{Mag}}}} F\left( x \right) = \;^{{{\text{Forcing}}}} F\left( {x,t} \right) + \;^{{{\text{Grav}}}} F\left( t \right)} \hfill \\ {V + L\dot{I} + R_{I} I + \alpha \left( x \right)\dot{x} = 0} \hfill \\ \end{array} } \right.$$

where $m$ is the mass of the levitating magnet, $^{{{\text{Damp}}}} F\left( {\dot{x}} \right)$ a damping force (usually dependent on the velocity), $^{{{\text{Grav}}}} F$ the gravity force ($\approx - mg\cos \left( \theta \right)$, $g$ the standard gravity acceleration) and $L$ and $R_{I}$ the equivalent inductance and resistance of the coils, respectively. The top equation results from the conservation of linear momentum of the system; the bottom one is the $V$–$Q$–$x$ relationship resulting from Faraday’s law and Kirchoff’s circuit laws. Assuming a viscous friction with mechanical damping coefficient $c = 2m\omega_{0} \zeta_{{\text{M}}}$ results in the simple form for the damping term: $^{{{\text{Damp}}}} F = - c\dot{x}$. Usually a resistive load ($R$) is connected to the system so that Ohm’s law applies: $V = RI$. A low-frequency approximation is also commonly considered, in which the $L\dot{I}$ term in the bottom equation is assumed to be small when compared to the other terms (i.e., for angular frequency of excitation $\omega \ll \left( {R + R_{I} } \right)/L$) and, thus, the current can be written explicitly as [37]: $I = - \alpha \left( x \right)\dot{x}/\left( {R + R_{I} } \right)$. This simplification shows that the current tends to be ca. proportional to the velocity of the moving magnet and so does the voltage, as can be verified in the time response of an EMG to a square-wave input shown in Fig. 4a (ii, iv), which is due to the resistive nature of the system at low frequencies. A harmonic input displacement $x$ with constant amplitude also results in an output voltage and current with amplitudes linearly increasing with the frequency, as can be observed in Fig. 4b (ii, iv). The current in this form can then be plugged into the top Eq. (7) resulting in a single second-order ODE. For harvesting systems with cylindrical symmetry, the Lorentz force produced by the coil on the levitating magnet turns out to be simply [155]: ${}_{ }^{Lrz} F\left( {x,I} \right) = \alpha \left( x \right)I$, which, together with the low-frequency approximation, results in an ODE with a load dependent electromechanical damping constant equal to [142]: $\alpha \left( x \right)^{2} /\left( {R + R_{I} } \right)$. In practice, this equation is still hard to solve analytically since the magnetic force and electromechanical coupling coefficient usually change highly nonlinearly with the position of the free magnet. Approximated solutions are commonly obtained analytically using the perturbation method of multiple scales [160] or numerically, e.g., using Runge–Kutta methods [155, 162, 163]. As an example, the magnetic force and electromechanical damping terms can be expanded in a power series around an equilibrium point. In first approximation, truncating the powers up to order one for the magnetic force ($\omega_{0} \left( x \right) \approx \omega_{0}$) and zero for the electromechanical coefficient ($\alpha \left( x \right) \approx \alpha_{0}$), and with an harmonic forcing term ($^{{{\text{Forcing}}}} F\left( t \right)/m = A \cdot \cos \left( {\omega t} \right)$ and $^{{{\text{Grav}}}} F\left( t \right) = 0$), results in the linear driven harmonic oscillator equation with a well-known analytical solution [164]. The corresponding complex displacement phasor (i.e., for a complex exponential response formula $z = \hat{x}e^{i\omega t}$), current phasor and average output power are given by the following equations [153, 155]:

8 $$\hat{x} = \frac{A}{{\left( {\omega_{0}^{2} - \omega^{2} } \right) + i\omega \omega_{0} 2\left( {\zeta_{{\text{M}}} + \zeta_{{{\text{EM}}}} } \right)}}$$

9 $$\hat{I} = \frac{{\hat{V}}}{R} = - \frac{{\alpha_{0} }}{{R + R_{I} }}i\omega \hat{x}$$

10 $$P = \frac{m}{{\omega_{0} }}A^{2} \frac{R}{{R + R_{I} }}\zeta_{{{\text{EM}}}} \frac{{\left( {\omega \omega_{0} } \right)^{2} }}{{\left( {\omega_{0}^{2} - \omega^{2} } \right)^{2} + \left( {\omega \omega_{0} 2\left( {\zeta_{{\text{M}}} + \zeta_{{{\text{EM}}}} } \right)} \right)^{2} }}$$

11 $$\zeta_{{{\text{EM}}}} = \frac{{\alpha_{0}^{2} }}{{R + R_{I} }}\frac{1}{{2m\omega_{0} }}.$$

These expressions show that all electrical parameters have a resonant behavior taking maximum absolute peak values for $\omega = \omega_{0}$ and the displacement, voltage and current increase linearly with the acceleration amplitude ($A$), while the average power increases with its square. Larger values of the load resistance ($R$) also correspond to smaller electromechanical damping ratio ($\zeta_{{{\text{EM}}}}$) and thus larger displacements and sharper peaks. For low values of the frequency (i.e., significantly lower than the resonance frequency), both the current and voltage can also be seen to increase ca. linearly with the frequency $\omega$. The EMG behaves broadly like an Thévenin/Norton equivalent circuit composed of a voltage/current source, with amplitude $\hat{V}_{{{\text{OC}}}}$/$\hat{I}_{{{\text{SC}}}}$ as given by Eq. (9) in the limit of an infinite/null load resistance $R$, and an internal impedance in series/parallel given by the ratio $Z = \hat{V}_{{{\text{OC}}}} /\hat{I}_{{{\text{SC}}}}$. More generally, truncating the powers up to order 3 in the expansion of Eq. (7) results in a form similar to a Duffing’s differential equation with a cubic nonlinearity [160, 165]. With a sinusoidal driving force, this equation reveals some of the characteristic behavior of the steady state response for a given frequency of excitation. Such a system typically exhibits an hardening stiffness, with the frequency response overhanging to the high-frequency side and a wider bandwidth, as well as an hysteretic response, with two solutions for frequencies close to the natural frequency, as well as amplitude jumps at two points [160, 165]. Part of this characteristic behavior can be observed in the frequency response of a nonlinear coupled magnet–spring–coil EMG and TENG hybrid structure shown in Fig. 7b (i, ii). In the general case, the nonlinearity of the electromechanical coefficient also results in higher harmonic output electrical signals with characteristic frequencies at multiples of the input frequency (Fig. 7b (iv)), each one initially increasing proportionally with higher powers of the acceleration amplitude.

The electrical behavior of EMGs is equivalent to that of TENGs as a function of the load resistances having three working regions. Low values of load resistance ($R \to 0$ in Eq. (9)) correspond to a large electromechanical damping and, consequently, to small maximum displacement and velocity amplitudes. The voltage $V$ is ca. null, and the current $I$ is maximized because of the large transfer rate of charge. Large values of the load ($R \to \infty$ in Eq. (9)) correspond to small values of electromechanical damping, resulting in large maximum displacements and velocities. As a result, the charge transfer rate is small, $I$ goes to 0 and *V* is maximized. The average electrical power output on the load attains a maximum in between these two points and for a matching resistance, which, according to the linear model under resonance conditions ($\omega = \omega_{0}$), is equal to the resistance of the coil plus a ratio between the square of the electromechanical coupling factor and the viscous friction mechanical damping coefficient (i.e., $R_{{\omega_{0} }} = R_{I} + \alpha_{0}^{2} /c = R_{I} + R_{I} \zeta_{{{\text{EM}}0}} /\zeta_{{\text{M}}}$, with $\zeta_{{{\text{EM}}0}} = \alpha_{0}^{2} /R_{I} 2m\omega_{0}$) [164]. The maximum average power peak extractable under resonance conditions, as in Eq. (6) with $\zeta_{{\text{E}}}$ separated into its external and internal load components [164], should also increase with the $mA^{2} /\omega_{0}$ power factor and ca. with the inverse of the mechanical damping ratio $\zeta_{{\text{M}}}$ (i.e., $\left\langle P \right\rangle_{{\omega_{0} ,\max }} = \left( {mA^{2} /\omega_{0} } \right)\left( {1/8} \right)\left( {1/2\zeta_{{\text{M}}} } \right)\left( {\zeta_{{{\text{EM}}0}} /\left[ {\zeta_{{{\text{EM}}0}} + \zeta_{{\text{M}}} } \right]} \right)$). However, we should note that for low values of $\zeta_{{\text{M}}}$, in practice the power is further limited by the maximum possible displacement of the moving parts inside the generator (given by Eq. (5)), while also the matching load for maximum power goes to infinity in the limit when $\zeta_{{\text{M}}}$ goes to zero. Furthermore, from Eq. (9), the OC voltage amplitude peak under resonance conditions can be shown to take the value $V_{{{\text{OC}}_{{\omega_{0} }} }} = \left( {mA} \right)\left( {R_{I} /\alpha_{0} } \right)\left( {\zeta_{{{\text{EM}}0}} /\zeta_{{\text{M}}} } \right)$, in general roughly increasing with the number of loops in the coil ($R_{I}$ and $\alpha_{0}$ roughly proportional to the number of loops) and the SC current amplitude $I_{{{\text{SC}}_{{\omega_{0} }} }} = \left( {mA} \right)\left( {1/\alpha_{0} } \right)\left( {\zeta_{{{\text{EM}}0}} /\left[ {\zeta_{{{\text{EM}}0}} + \zeta_{{\text{M}}} } \right]} \right)$, roughly decreasing with the number of loops. The values of the matching resistance for maximum output average power and corresponding average power can be related to these two parameters by: $R_{{\omega_{0} }} = V_{{{\text{OC}}_{{\omega_{0} }} }} /I_{{{\text{SC}}_{{\omega_{0} }} }}$ and $\left\langle P \right\rangle_{{\omega_{0} ,\;\max }} = \left( {1/8} \right)V_{{{\text{OC}}_{{\omega_{0} }} }} I_{{{\text{SC}}_{{\omega_{0} }} }}$. The energy conversion efficiency over one cycle with period $T$ in the steady state can be given by: $\eta = \mathop \smallint \limits_{0}^{T} VI{\text{d}}t/\mathop \smallint \limits_{0}^{T} - \left( {^{Lrz} F + \;^{{{\text{Damp}}}} F} \right)\dot{x}{\text{d}}t = R\zeta_{{{\text{EM}}}} /\left( {R + R_{I} } \right)\left( {\zeta_{{{\text{EM}}}} + \zeta_{{\text{M}}} } \right)$. Scaling each dimension of EMG by a constant $c$ term, while maintaining the aspect ratio, would result in the transformations: $\alpha \left( x \right) \to c \cdot \alpha \left( {x/c} \right)$, $^{{{\text{Mag}}}} F\left( x \right) \to c^{2} \cdot^{{{\text{Mag}}}} F\left( {x/c} \right)$, $\omega_{0} \to c^{ - 1} \cdot \omega_{0}$, $m \to c^{3} \cdot m$, $R_{I} \to c \cdot R_{I}$, and corresponding average powers for a matching load scaling with $c^{5}$, at low frequencies, $c$, at large frequencies, and ca. $c^{6}$, under resonance conditions.

For the general case of EMGs, transduction models usually make fundamental use of the quasi-magnetostatic and quasi-electrostatic forms of the Maxwell’s equations, Lorentz’s force and continuum mechanics [166, 167]. Modeling such complex multiphysical systems involves multiple steps namely the computation of body dynamics, magnetic fields, magnetic forces, electromotive forces, damping forces and electrical circuits [153]. Magnetic fields produced by the permanent magnets and currents flowing through the coils are calculated using Gauss’s law for magnetism and Ampère’s circuital law, and solving the resulting partial differential equations or its integral form obtained through the Helmholtz decomposition theorem (i.e., the Biot–Savart equation) to find the magnetic vector or scalar potentials. The forces between magnets and current loops can be calculated using the previously obtained magnetic fields. The continuous Lorentz force equation and the magnetic force between magnets can be obtained using equivalent surface current (Ampère model) [154, 168] or charge (Gilbert model) [168, 169] models or, alternatively, magnetic energy variation [155, 170] or Maxwell’s stress tensor methods [171, 172]. For the special case of a system with cylindrical symmetry, the magnetic fields and forces can be analytically computed using elliptic integrals [155, 173, 174] or Bessel functions [155, 161]. For more complicated geometries the equations cannot be analytically solved, so numerical methods (such as FEM) can be used [175–179]. Electromotive forces are determined from the magnetic fields and Faraday’s law of induction and usually calculated by integration and superposition for single loops of wire [37, 154, 155]. In the case of the damping forces, various friction models have been considered containing several components including constant dry friction opposing the movement direction, viscous friction proportional to the velocity and skin drag friction proportional to the square of the velocity, with the corresponding damping constants being obtained empirically [155, 156, 163]. After obtaining the relationship between electrical and dynamical parameters for the generators, equivalent electrical networks with external components are modeled using Kirchoff’s circuit laws [155, 158, 161, 170].

## Design Configurations and Applications of Hybrid E-TENGs

The design configuration of hybrid E-TENGs is of upmost importance as it significantly influences the efficiency of generation, type of mechanical input (rotation, translational or arbitrary) and range of operation of input amplitudes and frequencies. Generally, TENGs take vertical contact-separation, lateral-sliding, single-electrode or free-standing structures [1, 3, 111]. Developed EMGs have been widely composed by one or more NdFeB magnets with square, cylindrical or sphere shapes and neighbor pickup coils, mostly with helix or spiral shapes. Both systems have been integrated using different architectures and aimed at different types of mechanical inputs, including for rotating, linear and multidimensional motions.

### Rotating Generators

**Fig. 8:**
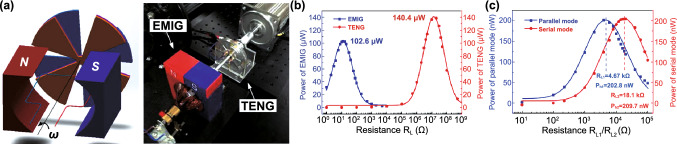
**a** Schematic diagram and photograph of the original E-TENG hybrid generator developed by Zhang et al. **b** Output average power of the rotating DC-EMG and DC-TENG cells with different external resistive load. **c** Output power of the combined generators connected to impedance matching resistors and in parallel and series between themselves. Reproduced with permission from Ref. [63]. Copyright 2014, Advanced Materials. (Color figure online)

This section analyzes eleven hybrid E-TENG technologies based on rotational architectures. One of the first systematic studies establishing the theoretical and experimental foundation of the hybridized operation of TENG and EMG cells for harvesting mechanical energy was performed by Zhang et al. [63] in 2014. As depicted in Fig. 8a, an hybrid EMG/TENG generator was fabricated with the sliding-mode Kapton/Al TENG, consisting of two segmentally patterned disk-shaped layers rotating in relation to each other [180], while the EMG consisted of a group of coils rotating around the central axis inside a pair of permanent magnets. The TENG was shown to behave ca. like an ideal current source in parallel with a high internal impedance, and the EMG similar to an ideal voltage source in series with a low internal impedance. The SC output current of the TENG was observed proportional to the triboelectric charge density, while the OC output voltage of the EMG was proportional to the magnetic flux density, with both also increasing with the generator’s size and input angular velocity. Operating independently with the same angular velocity input of 600 rpm, the TENG cell generated output rectified powers up to 140.4 µW for a large optimal load of 13.8 MΩ and the EMG up to 102.6 µW for a small matching load of 12.3 Ω, as shown in Fig. 8b. Direct electrical connection of both cells in series and parallel resulted in output powers similar to those of the individual current source TENG and voltage source EMG, respectively, due to the internal impedance mismatch. To solve this coupling problem, a simple power transformation design was experimented in which the rectified TENG was connected with an additional resistor in parallel and the rectified EMG with the same resistor in series, thus bringing together the optimal load resistance of both devices to ca. 9.43–9.77 kΩ and corresponding individual output DC power to 117.6–118.1 nW. Connecting the generators in series and parallel thus yielded a response with the previous maximum output powers ca. adding up in linear superposition as depicted in Fig. 8c.

**Fig. 9:**
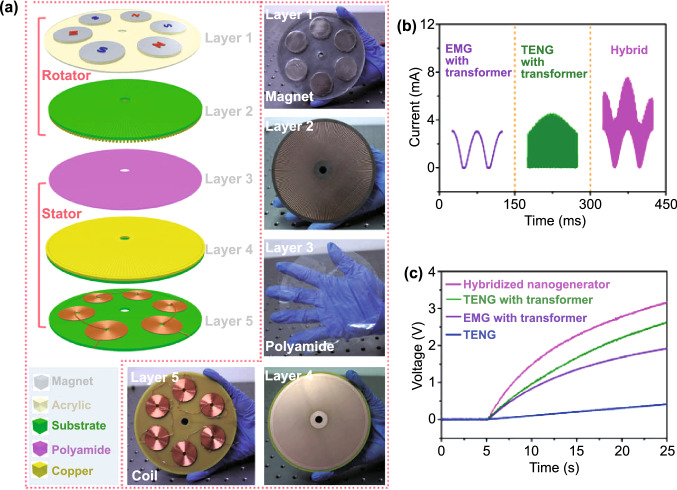
**a** Schematic diagram and photographs of the rotating-disk-based hybridized EMG-TENG nanogenerator developed by Zhong et al. **b** Comparison of the SC current output for the EMG with transformer, TENG with transformer and the hybridized device. **c **Measured voltages across a 6600 μF capacitor charged by the TENG, TENG/EMG with transformer and the hybridized nanogenerator (EMG and TENG with transformer in parallel). Reproduced with permission from Ref. [64]. Copyright 2014, Elsevier Ltd. All rights reserved. (Color figure online)

Likewise, Zhong et al. [64] designed a more compact rotating-disk-based hybridized nanogenerator for scavenging biomechanical energy. This consisted of a rotator disk with 140 mm of diameter, six evenly spaced magnets and a stator disk with six spiral-shaped coils together with a lateral-sliding TENG made of polyamide and radial-arrayed (ca. 1º spaced) metal Cu strips (see Fig. 9a). In this rotating system, the output electrical signals have dominant components at the frequency of rotation times half the number of generator units around the circumference. Thus, in this study the working frequency of the TENG was 60 times larger than that of the EMG, as can be perceived in Fig. 9b. For a rotation rate of 200 rpm, the TENG and EMG units were shown to generate OC voltages with peak values up to 75 and 0.62 V, respectively, SC currents of 0.33 and 57.8 mA and maximum powers of 8.6 and 8.4 mW for optimal loads of 0.2 MΩ and 12Ω. To solve the impedance mismatch issue, two transformers were used to decrease and increase the equivalent internal impedances of the TENG and EMG, respectively. The output characteristics thus changed to 5.5 V and 3.87 mA for the TENG and 4.3 V and 4.3 mA for the EMG with corresponding matching loads of the order of 1 kΩ, although this also introduced additional power losses associated with the transformers. Further using two bridge rectification circuits to convert the AC signals of the TENG and EMG, connected in parallel, into DC output, an impedance matching of the TENG and EMG was achieved with the output current signals adding up linearly up to a maximum peak of ca. 8 mA, as show in Fig. 9b. The hybridized nanogenerator was also shown to have a significantly better capacitor charging performance than that of the individual energy harvesting cells, as can be seen in Fig. 9c. A prototype device of a human hand-driven hybridized nanogenerator was fabricated and tested as a mobile power source to continuously light up a group of LEDs and a white globe light.

**Fig. 10:**
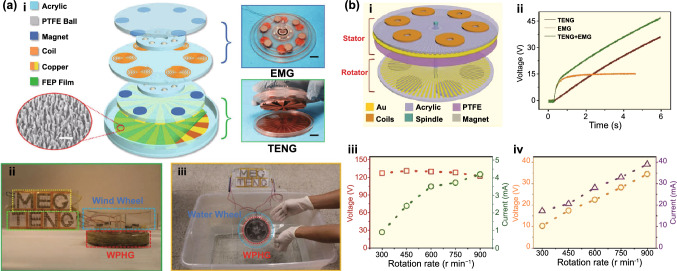
**a** (i) Structure design of the waterproof hybrid E-TENG generator fabricated by Guo et al. (ii) Demonstration of the generator for harvesting wind energy under simulated rainy condition. (iii) Generator used for harvesting water flow energy under water. Reproduced with permission from Ref. [65]. Copyright permissions from 2016, Advanced Energy Materials. **b** (i) Diagram of the rotating-disk-based hybridized nanogenerator designed by Chen et al. (ii) Charging performances of the TENG, EMG and hybridized nanogenerator for a 470 μF capacitor. Variation of the output OC voltage and SC current with the rotation rate for the (iii) TENG and (iv) EMG. Adapted from Ref. [66]. (Color figure online)

A similar hybrid disk-shaped encapsulated waterproof triboelectric-electromagnetic hybrid generator (WPHG) with ball separated sliding free-standing layers, as depicted in Fig. 10a (i), was developed by Guo et al. [65]. The TENG was shown to generate a constant voltage output and a current linearly increasing with the rotation speed; differently, the EMG generated voltages and currents linearly increasing with the rotation speed. Thus, results demonstrated the superiority of the TENG operating under low-frequency conditions. Under a rotation speed of 1600 rpm, the obtained maximum output voltage, current and power were: ca. 500 V, 100 µA and 7 mW at 10 MΩ for the TENG; 2.9 V, 15 mA and 4.5 mW at 60 Ω for the EMG. Using transformers, the matched resistances of the TENG and EMG were adjusted to 1 and 1.2 kΩ, respectively, and, consequently, the OC voltages were both adjusted to ca. 5 V and the SC currents changed to 1.5 and 1.2 mA. With a full-wave rectifier and both units connected in parallel, a supercapacitor (20 mF) was charged up to 1 V in 22 s. The encapsulation of the hybrid system allowed it to be shielded from the negative effects of harsh environmental conditions and it was thus demonstrated harvesting wind energy under rainy conditions and water flow energy under water (Fig. 10a (ii, iii)).

Chen et al. [66] designed another large rotating-disk-based hybridized nanogenerator to simultaneously harvest rotational energy and wirelessly transmit power. The sliding free-standing TENG consisted of radially arrayed Au sectors in the rotator and a PTFE stator and the EMG of six magnets and coils integrated in the rotator and stator, respectively (Fig. 10b (i)). For an increasing rotation rate, the voltage output of the TENG unit was shown to remain unchanged and the current to increase linearly, while the voltage and current output of the EMG simultaneously increased linearly, as depicted in Fig. 10b (iii, iv). Under a rotation rate of 900 rpm, the EMG produced output powers up to 137.39 mW at a matched load resistance of 300 Ω, and the TENG up to 217.8 mW at 20 kΩ. The outputs of both units were connected to two bridge rectification circuits and used to charge a 470 μF capacitor, resulting in a faster charging determined by the higher output current of the EMG and a larger final voltage dominated by the output voltage of the TENG (Fig. 10b (ii)). A commercial transformer was also adopted to increase the output current of the TENG, further improving the charging speed of the hybridized nanogenerator. Furthermore, the power from the generator was wirelessly transmitted to a distance of ~ 60 cm in real time based on helical coils.

**Fig. 11:**
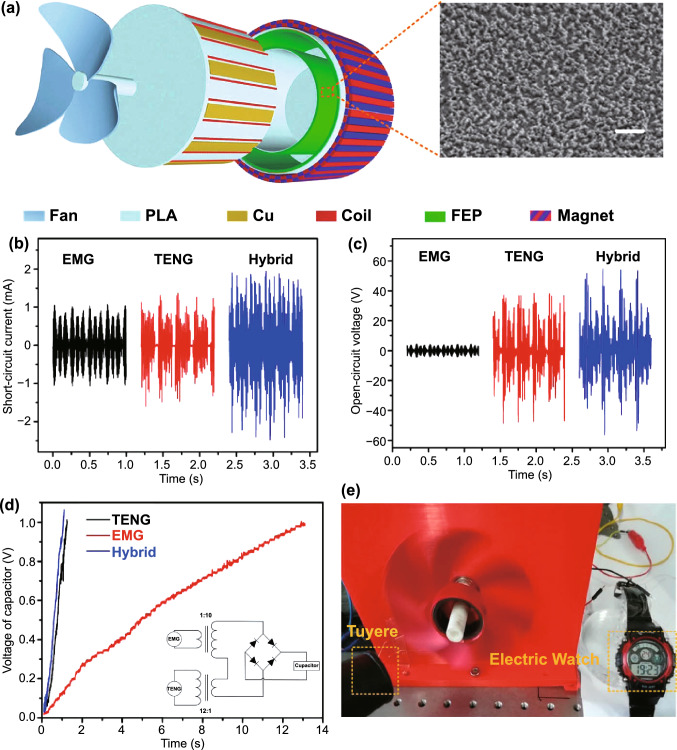
**a** Structure of the rotating-sleeve hybrid E-TENG generator nanogenerator and SEM image of the FEP film nanostructure (scale bar, 2 μm) engineered by Cao et al. **b** SC current of the EMG, TENG and hybrid with transformers when connected in parallel. **c** OC voltage of the EMG, TENG and hybrid with transformers when connected in series. **d** Charging curves of a 470 μF capacitor by the TENG, EMG and hybrid and equivalent rectifier circuit diagram. **e** Demonstration of an electronic watch powered by the hybrid generator under blowing wind from a tuyere. Reproduced with permission from Ref. [67]. Copyright 2017, American Chemical Society. (Color figure online)

Cao et al. [67] engineered a wind-driven rotating-sleeve-based hybrid nanogenerator for converting mechanical energy into electric energy with high conversion efficiency. The device structure is schematically represented in Fig. 11a. It comprises a stator and a rotator with the sliding free-standing TENG, composed of a nanostructured FEP film and a grating-structured Cu electrode, and the EMG, composed of alternating radially magnetized permanent magnets and coils intertwined in the grooves of the torus. Impedance matching was achieved through a commercial step-down transformer in the TENG, adjusting the output characteristics from 120 μA and 580 V to 1.5 mA and 50 V, and step-up transformer in the EMG, changing the output characteristics from 11.5 mA and 0.85 V to 1 mA and 7 V. The matched resistance of the TENG exhibits a decreasing tendency with increasing rotation speed, due to constant OC voltage and increasing SC current. Transformers must be appropriately chosen considering this fact. Compared to the individual generators, the E-TENG hybrid generator, connected in parallel/series, yielded an added current/voltage output as shown in Fig. 11b, c. For 400 rpm, the maximum SC current was 2.2 mA and OC voltage of 48 V, while a power up to ca. 13 mW was achieved for an 8 kΩ matched load resistance. With an added rectifier, as illustrated in the equivalent circuit in the inset of Fig. 11d, the hybrid generator also yielded a slightly faster capacitor charging time. With an added fan, the device was shown to be capable of lighting dozens of LEDs as well as powering an electronic watch under blowing wind (Fig. 11e).

**Fig. 12:**
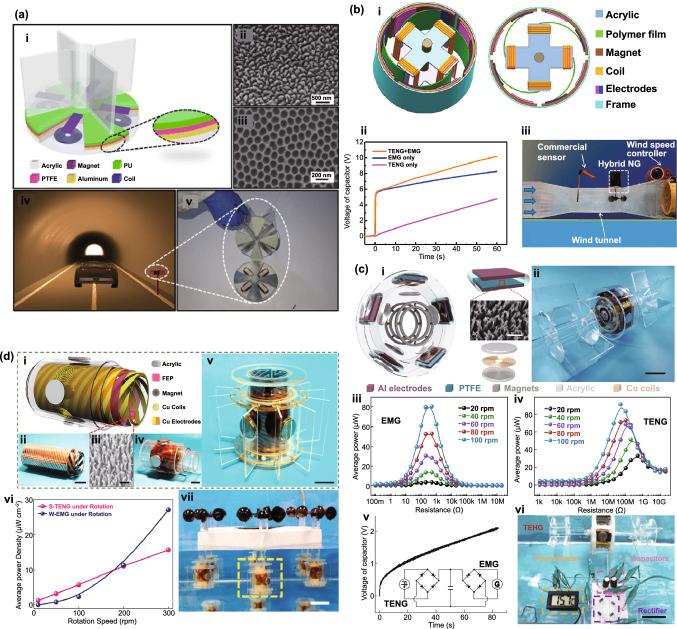
**a** Structural design of the rotating-disk-based hybrid generator for self-powered wireless traffic volume sensing developed by Zhang et al. (i) Sketch of the generator. SEM image of the (ii) PTFE surface with nanostructures and (iii) aluminum surface with nanopores. (iv) Schematic diagram of the generator working in a tunnel. (v) Photograph of the self-powered sensing system. Reproduced with permission from Ref. [68]. Copyright permissions from 2016, American Chemical Society. **b** (i) Schematic illustration of the ultra-low-friction wind energy hybrid nanogenerator developed by Wang et al. (ii) Charging curve of a 100 μF capacitor by the TENG, EMG and hybrid generator. (iii) Demonstration of the ultra-low-friction hybrid nanogenerator for energy scavenging and self-powered wind speed sensing in a wind tunnel. Reproduced with permission from Ref. [69]. Copyright permissions from 2018, American Chemical Society. **c** (i) Schematic illustration and (ii) photograph of the blue energy hybrid generator, consisting of five contact-separation-mode TENGs and five rotary free-standing-mode EMGs, designed by Shao et al. Output average power of the (iii) EMG and (iv) TENG as a function of the load resistance and rotation rate. (v) Measured voltages of a 33 μF capacitor charged by the hybrid generator. (vi) Photograph of the self-powered water temperature sensing system, including rectifiers, capacitors and an electronic thermometer. Reproduced with permission from Ref. [70]. Copyright 2018, American Chemical Society. **d** (i) Schematic illustration of the functional components of the hybrid nanogenerator for harvesting broad frequency band blue energy developed by Wen et al., mainly consisting of a spiral-interdigitated-electrode TENG and a wrap-around EMG. Photographs of (ii) an as-fabricated TENG and (iv) an as-fabricated EMG (scale bars are 2 cm). (iii) SEM image of the FEP polymer nanowires in the TENG (scale bar is 500 nm). (v) Digital photograph of an as-fabricated hybrid nanogenerator. (vi) Optimized average power densities of the TENG and EMG operated under rotation. (vii) Photograph of the proposed comprehensive energy harvesting panel floating on the ocean for simultaneously harvesting wind, solar and wave energies. Reproduced with permission from Ref. [71]. Copyright 2016, Creative Commons Attribution 4.0 International License. (Color figure online)

A rotating-disk-based generator for sustainably powering wireless traffic volume sensors by harvesting energy from the wind generated by a moving vehicle through a tunnel was devised by Zhang et al. [68] (Fig. 12a). The rotator was composed of four acrylic intercrossing blades, anchored on a disk acrylic substrate with four nanostructured PTFE layers and bar magnets, and the stator of an acrylic base with four Al electrodes and coils. To solve the critical impendence mismatch issue of the TENG and EMG, a transformer was employed to decrease the impendence of the TENG, and two bridge rectification circuits were utilized to convert the alternating current (AC) into direct current (DC). At a load resistance of 700 Ω, an optimized instantaneous power of 17.5 mW was obtained under 1000 rpm.

An ultra-low-friction hybrid nanogenerator for small external force energy harvesting and self-powering sensors was developed by Wang et al. [69]. As seen in Fig. 12b (i), it consisted of a cylindrical stator with four arc-shaped magnets and a rotator with a cross-shaped vertical structure with four groups of coils. The sliding free-standing TENG was implemented with four elastic blades made of soft flexible FEP, PTFE or Kapton thin films in the rotator and Cu electrodes in the stator. These soft contacts resulted in very small friction forces. Testing was performed for different TENG materials, dimensions and rotation speeds. The output voltage of the TENG was shown to increase with the rotation speed due to the increase of the contact area between the low-friction blades and electrodes. A maximal power per unit of mass of 181 mW kg^−1^ at 80 Ω was obtained for the EMG and 439 mW kg^−1^ at 40 MΩ for the TENG. As shown in Fig. 12b (ii), the EMG was observed to charge a 100 μF capacitor faster than the TENG, although only up to a smaller voltage of ca. 6 V, which is related to the high output current characteristics of the former and high output voltage of the latter. The hybrid device charged the capacitor ca. as a linear superposition of the output from the individual generators. This hybrid generator was also demonstrated powering a humidity/temperature sensor and a self-powered wind speed sensor by converting wind energy into electric energy (Fig. 12b (iii)).

Shao et al. [70] designed a fully packaged hybrid generator for harvesting water flow-based blue energy, including multiple separation-mode triboelectric nanogenerators and rotary free-standing-mode electromagnetic generators operated by magnetic repulsion. As depicted in Fig. 12c (i, ii), five coils were arranged on the outer surface of a cylinder with five TENGs, made of nanowired PTFE and Al films, fixed on each coil. The TENGs had one magnet at the other end and counterpart magnet in the inner rotating tube containing two impellers at both ends. The working mechanism is based on the periodic magnetic repulsion between aligned magnets and corresponding change in magnetic flux and displacement. For a top rotation speed of 100 rpm, the TENG’s output OC voltage, SC current and maximum average power were up to 315.8 V, 44.6 µA and 90.7 µW, respectively, and the outputs of the EMGs were up to 0.59 V, 1.78 mA and 79.6 µW, respectively (Fig. 12c (iii, iv)). The hybrid device was demonstrated powering LEDs and charging commercial capacitors, as well as using the stored energy to power an electronic water thermometer (Fig. 12c (v, vi)).

Another packaged hybrid nanogenerator, consisting of a spiral-interdigitated electrode triboelectric device and a wrap-around electromagnetic generator, was developed by Wen et al. [71] to harvest broad frequency band blue energy. The basic structure of the generator, depicted in Fig. 12d (i–v), comprise three coaxial acrylic cylindrical tubes, in which the rotating inner one and fixed middle one containing a spiral-shaped free-standing TENG is composed of a foam and a FEP thin film with nanowire and deposited electrode Cu stripes. The EMG was engineered with four pairs of magnets, evenly fixed on the inside surface of the rotating inner tube and outside surface of the rotating outer tube, and four Cu synclastic twined coils embedded in the outside surface of the middle tube. The spiral-interdigitated-electrode shape allowed to feature the TENG with dual-function: to harvest both rotational and fluctuation energies (i.e., for translations along the axial direction). Under rotation and vibration conditions, the output average power density from the TENG was shown to increase linearly with the rotation rate and frequency, while the power from the EMG increased with its square, surpassing the performance of the former only for values larger than ca. 200 rpm and 3 Hz (Fig. 12d (vi)). Thus, the output power could be adjusted for a more efficient energy harvesting over a broader frequency range. Under a rotation rate of 300 rpm or a displacement of 20 mm with 5 Hz of frequency, the TENG provided average power densities up to ca. 15.67 and 4.07 μW cm^−2^, respectively, and the EMG provided up to ca. 27.12 and 6.33 μW cm^−2^. An applied energy harvesting panel floating on the ocean was proposed as illustrated in Fig. 12d (vii), consisting of six wind and water flow driven nanogenerator units with added rotor blades and solar cell panels.

**Fig. 13:**
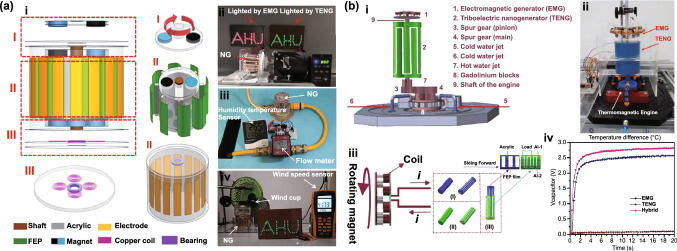
**a** (i) Schematic diagram of the easily assembled hybrid nanogenerator designed by Zhong et al., including a magnetic coupling (I), sliding free-standing mode TENG (II) and EMG (III). Demonstrations of the hybrid generator for various applications: (ii) LEDs lit by the individual EMG and TENG driven by a motor. (iii) Water flow energy harvested to power a humidity/temperature sensor. (iv) Wind energy harvested to light LED arrays and power a wind speed sensor at a wind speed of 4 m s^−1^. Reproduced with permission from Ref. [72]. Copyright permissions from 2019, WILEY‐VCH Verlag GmbH & Co. KGaA, Weinheim. **b** Thermomagnetic magneto‐caloric hybrid EMG/TENG generator developed by Ahmed et al. (i) Scheme and (ii) photograph of the generator. (iii) Detail of the rotating EMG (left) and TENG (right). (iv) Charging curves of a 1000 μF capacitor by the rectified output of the EMG, TENG and hybrid. Reproduced with permission from Ref. [73]. Copyright 2019, John Wiley & Sons, Ltd. (Color figure online)

An easily assembled hybrid nanogenerator driven by magnetic coupling for fluid energy harvesting and self-powered flow monitoring in a smart home/city was developed by Zhong et al. [72]. The structural design of the hybrid nanogenerator is shown in Fig. 13a (i). It comprises a cylindrical stator and a cylindrical rotator. The latter contains: several pieces of flexible FEP films on the sides and two driving magnets, with different magnetic pole directions, embedded in the upper cover; four magnets of the EMG, with identical magnetic pole directions, embedded in the lower cover. The stator contains several pieces of Cu foils and four Cu coils fixed on the inner surface of the lower cover. The effect of the rotation speed on the output performance was studied: the TENG provided a maximal load power of 1.05 mW for a resistance of 4 MΩ; and 58.3 mW at a matched load of 20 Ω, for the EMG, at a rotation speed of 500 rpm. The nanogenerator could be conveniently integrated with a driving wind cup or water turbine and was demonstrated harvesting fluid energy to power small electronic devices, such as a humidity sensor and a temperature sensor while being driven by a motor, tap water flow and fan wind flow (Fig. 13a (ii–iv)).

Ahmed et al. [73] devised a new thermomagnetic generator, based on the magneto‐caloric effect of Gadolinium, to scavenge low‐grade thermal energy through a hybrid EMG/TENG structure. As depicted in Fig. 13b (i–iii), the cylindrical-shaped rotor of the generator consists of 16 Gadolinium blocks and a bar‐shaped permanent magnet that rotates in response to hot and cold water jets impinging on the Gadolinium blocks, changing their magnetic state between ferromagnetic and paramagnetic. The rotational motion energy was then converted into electrical energy through a cylindrical sliding-mode free-standing FEP/Al TENG and a disk‐shaped EMG with eight magnets and coils (Fig. 13b (iii)). The EMG generated AC output powers up to 15.62 mW at a load of 100 Ω, while the TENG provided 14.4 μW at a load of 10 MΩ. A pair of bridge rectifiers was used to combine the power outputs from the two different generators, delivering a maximum DC power output of 12.1 mW at 120 Ω. The proposed hybrid energy harvester is able to charge a capacitor slightly faster than the independent EMG and TENG (Fig. 13b (iv)), as well as to light dozens of LEDs or power a digital thermometer. The major advantage of this TENG here used is its non-magnetic nature and, thus, its insusceptibility to the strong magnetic field generated by the major components of the engine.

### Pendulum Generators

**Fig. 14:**
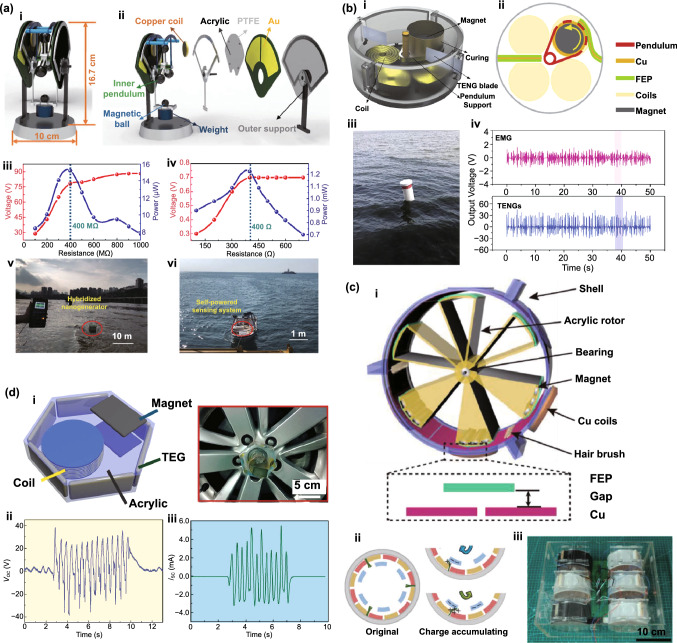
**a** (i) Structural schematic diagram and (ii) exploded view of the chaotic pendulum hybridized nanogenerator designed by Chen et al. Dependences of the output voltage and power of the (iii) TENG and (iv) EMG with the load resistance. (v) Hybridized nanogenerator being tested in a lake. (vi) Self-powered wireless sensing node transmission system enabled by the hybridized nanogenerator in a boat. Reproduced with permission from Ref. [74]. Copyright 2019, Elsevier Ltd. All rights reserved. **b** (i) Schematic illustration and (ii) top view of the structure diagram of the rotational pendulum hybrid generator developed by Hou et al. (iii) Photograph of the hybrid generator inside a buoy being tested in a lake and (iv) output voltage of the EMG and TENG units when horizontally placed. Reproduced with permission from Ref. [75]. Copyright 2019, Elsevier Ltd. All rights reserved. **c** (i) Schematic illustration of the soft-contact cylindrical hybrid nanogenerator developed by Feng. et al. (ii) Working principle of the free-standing TENGs with FEP/Cu layers and rabbit hair brushes. (iii) Photograph of the hybrid array device with 6 units demonstrated in hydrological monitoring by water wave energy harvesting. Reproduced with permission from Ref. [76]. Copyright 2021, Nano Energy. **d** (i) Schematic illustration and photograph of the hybrid E-TENG tested by Fan et al. It is composed by six TENGs and one EMG units fixed to an automobile tire. (ii) Output OC voltage and (iii) SC current measured during rotation of the tire. Adapted from Ref. [77]. (Color figure online)

This section analyses four hybrid E-TENG technologies based on pendulum architectures. A chaotic pendulum triboelectric-electromagnetic hybridized nanogenerator for wave energy scavenging and self-powered wireless sensing system was developed by Chen et al. [74]. Owing to the randomness and low frequency of water waves, the nanogenerator was designed as an highly responsive chaotic pendulum consisting of an inner pendulum with three magnetic balls equally spaced on the rotating shaft and a major driving pendulum with a tip weigh (Fig. 14a (i, ii)). The free-standing TENG was made up of a PTFE film and two Au electrodes fixed onto the sector of the central pendulum. The EMG comprises three magnetic balls of the inner pendulum and three coils pasted on the inside acrylic installed on the sector of the central swing. The TENG reached 15.21 µW of instantaneous peak power output at 400 MΩ; the EMG reached higher instantaneous peak powers, namely 1.23 mW at 400Ω, as depicted in Fig. 14a (iii, iv). The harvester was also tested under real life conditions inside a buoy in a river (Fig. 14a (v)), as a part in a self-powered wireless sensing node system composed of a temperature sensor, radio-frequency (RF) module transmitter, RF receiver and monitoring system (Fig. 14a (vi)).

A rotational pendulum-based hybrid generator for low-frequency (< 5 Hz) multidirectional vibrations aiming at human motion and blue energy applications was developed by Hou et al. [75]. The design schematic of the hybrid nanogenerator is shown in Fig. 14b (i, ii). It includes a cylindrical frame with a pendulum rotor composed of a disk-shaped magnet and a Cu external ring, four disk coils fixed to the frame and two FEP flexible blades forming a contact-separation TENG module together with the Cu pendulum. The EMG reached a maximum instantaneous peak output power of 265 mW for a resistance of 75 Ω. Differently, the TENG provided 0.65 mW with a load of 10 MΩ at a driving frequency of 2 Hz and amplitude of 10 cm. Connecting the two modules to a rectifier and in parallel, the hybrid generator was able to charge a 22 µF capacitor, combining the faster charging speed from the EMG and higher charging level from the TENG. The hybrid generator was held in a tester's hand and mounted on the waist and tested by running and rope skipping, producing output voltages up to 9 V and 150 V for the EMG and TENG, respectively. It was also tested inside a sealed buoy in a lake, generating peak-to-peak OC voltages up to 3.4 V and 80 V for the EMG and TENG, respectively, regardless their orientation (Fig. 14b (iii, iv)).

A soft-contact cylindrical hybrid nanogenerator based on a swing structure for efficiently harvesting ultra-low-frequency water wave energy was recently developed by Feng et al. [76]. As schematically shown in Fig. 14c (i), the device consists of a stator containing 12 Cu electrodes and 3 tufts of dried rabbit hair in its interior surface and a stacked vane rotor with 6 FEP films glued onto the external surface. Free-standing TENGs were thus formed between non-contacting FEP and Cu layers with the hair brushed generating triboelectric charges when contacting with the FEP (Fig. 14c (ii)). This flexible rabbit hair brushes greatly reduces the operation friction resistance of the structure, enhances the durability and solves the charge dissipation problem. The EMG part was formed by 3 Cu coils installed onto the external wall of the shell and 3 groups of neodymium magnets embedded into the bottom part of acrylic rotor. Under water wave excitation with a wave height of 10 cm and frequency of just 0.1 Hz, a multiplied output frequency was observed with about 60 current pulses. Peak OC voltages of ca. 640 V, SC currents of 4.57 μA and instantaneous peak powers of 1.29 mW at a matched resistance of 150 MΩ were achieved for the TENG and 2.9 V, 11.9 mA and 3.5 mW at 300 Ω, for the EMG, respectively. The hybrid system was implemented by connecting the output terminals of the TENG and EMG in parallel with its output, which is essentially equal to the summation of the individual performances indicating no mutual interference. An array of hybridized nanogenerators was fabricated (Fig. 14c (iii)) and used for hydrological data monitoring and wireless transmission based on the ultra-low-frequency water wave energy.

Fan et al. [77] developed an hybrid rotating generator composed of one EMG unit and six contact-separation PDMS/ITO TENG units, both fixed to an automobile tire. As shown in Fig. 14d (i), this architecture consisted of a hexagonal prism container together with a permanent magnet, a mobile cylindrical mass with a coil and TENG units fixed to the walls. Due to their large internal impedance mismatch, the signals of TENGs and EMGs could not be enhanced simply by series or parallel connections. Thus a power transformation and managing system [181] was used both: to increase the current output of the TENG to match the output characteristics of the EMG; and to enhance the output performance of the hybrid structure beyond that of the individual cells. The hybrid generator thus yielded high OC voltage peaks up to 40 V and SC currents up to 5 mA (Fig. 14d (ii, iii)).

### Linear Generators

**Fig. 15:**
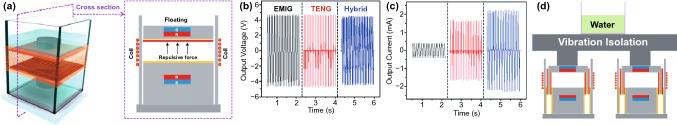
**a** Schematic diagram of the unidirectional hybrid cell integrated with a contact-mode TENG and EMG developed by Hu et al. and cross section view. After impedance match, **b** output voltage and **c** output current of the EMG/EMIG, TENG and hybrid cell (parallel connected EMG and TENG) on a load of 5 kΩ. **d** Diagram of the construction of the hybrid cell working as a both a vibration isolator and energy harvester. Reproduced with permission from Ref. [47]. Copyright 2014, American Chemical Society. (Color figure online)

Sixteen hybrid E-TENG technologies were already proposed based on linear architectures. To harvest unidirectional vibrational energy through multiple approaches, Hu et al. [47] designed an hybrid device consisting of two hollow cubes, one fixed and another floating, each with oppositely poled magnets surrounded by a coil and with the contact surfaces containing two sliding-mode Kapton/Cu TENGs or one single contact-separation TENG between the top and bottom layers (Fig. 15a). In this last scheme, the output signals from both the TENG and EMG were in phase and could thus be constructively added. Transformers (with a working frequency of 60 Hz) were used to match the output impedance between the two power sources and adjust the output voltages to a similar level. For a matched load of 5 kΩ, the output peak voltages of the TENG, EMG and hybrid cell were of ca. 4.6 V in all of them, the currents were of 1.6 0.4 and 2.2 mA, respectively, and the corresponding power peaks of up to 7.7, 1.9 and ca. 10 mW (Fig. 15b, c). A logic operation was also demonstrated on a half-adder circuit with the hybrid cell working as both the power source and input digital signals, as well as a vibration damper with integrated energy harvesting capabilities (Fig. 15d).

**Fig. 16:**
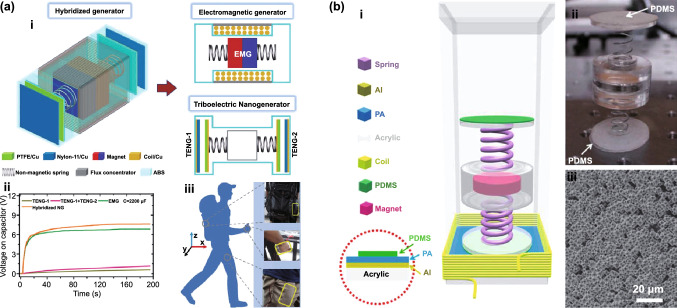
**a** (i) Structure of the elastic-impact-based non-resonant hybridized generator developed by Rahman et al. (ii) Capacitor charging characteristics of the TENGs, EMG and hybridized generator under 1 g of acceleration at 6 Hz. (iii) Demonstration of the fabricated prototype placed on the side-pocket of a backpack, trousers pocket or hand and under various human body-induced vibrations. Reproduced with permission from Ref. [78]. Copyright 2020, WILEY‐VCH Verlag GmbH & Co. KGaA, Weinheim. **b** (i) Schematic diagram of the hybrid energy cell studied by Wu et al. (b) Photograph of the hybrid energy cell. (ii) Photograph of the mass in the device. (iii) SEM image of the PDMS film. Reproduced with permission from Ref. [79]. Copyright 2014, Elsevier Ltd. All rights reserved. (Color figure online)

A biomechanical energy-driven elastic-impact-based non-resonant hybridized generator for universal powering was developed by Rahman et al. [78]. A detailed schematic of the E-TENG is illustrated in Fig. 16a (i). It comprises a rectangular hollow tube with a wrapped around coil and a freely moveable magnetic mass-based EMG attached to two nanowire-nanofiber PTFE/nylon contact-mode-based TENGs at both ends. The coil was covered with a soft magnetic material (SMM)-based flexible flux-concentrator film (FeSiCr/Ecoflex) to increase the induced electromotive force up to 1.39 times. Under a frequency of 6 Hz and acceleration of 1 g, maximal output powers of 142.42 mW at 76 Ω and 1.21 mW at 9 MΩ were obtained for the EMG and each TENG, respectively. Three bridge rectifier integrated circuits were used to combine the electrical output of each generator resulting in a maximum measured power of 144.1 mW with a voltage of 13.15 V at a matching load resistance of 1.5 kΩ. This design was useful for several applications such as charging a storage unit, powering commercial LEDs and temperature/humidity sensors from different human motions (Fig. 16a (iii)). The generator was also able to charge a 2200 µF capacitor in ca. 20 s (Fig. 16a (ii)) and, under human motion testing, generating an optimal output power up to 131.4 mW with horizontal handshaking.

Wu et al. [79] studied a similar hybrid energy cell for harvesting mechanical energy from one’s motion. As depicted in Fig. 16b (i, ii), it consists of an acrylic tube incorporating: an EMG that includes a floating magnet with two springs on the sides and a surrounding coil; and a contact-separation single-electrode TENG that includes a polydimethylsiloxane (PDMS) film with pyramid nanostructures (Fig. 16b (iii)) and a polyamide (PA) film. The largest output power provided by the EMG was 0.58 mW at 10 kΩ, while the TENG provided 0.25 mW at 100 MΩ. The hybrid generator was also used to directly power tens of LEDs and shown to have a better capacitor charging performance than the one obtained by individual generators under vibrations and height dropping.

**Fig. 17:**
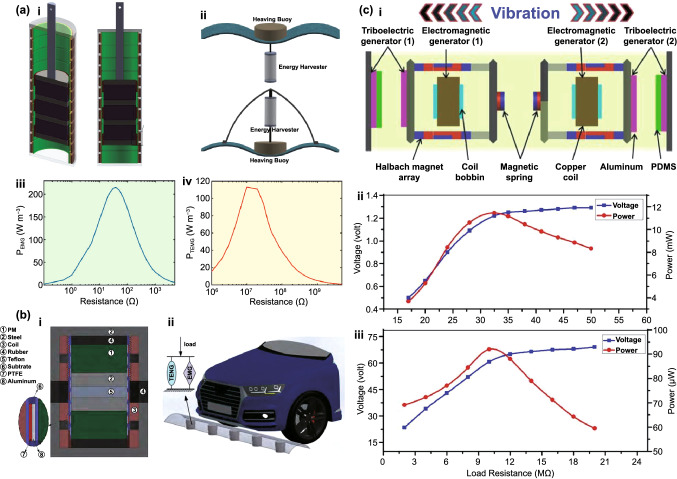
**a** (i) Heaving point absorber-based E-TENG wave energy harvester modeled by Saadatnia et al. (ii) Schematic model of the energy harvester integrated with an heaving buoy under water wave excitations. Calculated output power density as a function of the load resistance for the (iii) EMG and (iv) TENG. Adapted from Ref. [80, 81]. **b **(i) Cross section view of the hybrid generator proposed by Askari et al. (ii) Car passing over a speed bumper equipped by a hybridized nano generator. Reproduced with permission from Ref. [82]. Copyright 2016, Published by Elsevier Ltd. **c **(i) Schematic structure of the Halbach array-based hybrid energy harvester proposed by Salauddin et al. Load voltages (peak to peak) and powers (peak) versus load resistances generated by the (ii) EMG and (iii) TENG. Reproduced with permission from Ref. [83]. Copyright 2016, Creative Commons Attribution 3.0 license. (Color figure online)

A heaving point absorber-based wave hybrid energy harvester was modeled and analyzed by Saadatnia et al. [80, 81]. As shown in Fig. 17a (i, ii), the device comprises two main parts, namely a cylindrical stator and a slider. The slider is connected to a floating element buoy, such that is can be directly driven by wave force, and to a tubular permanent magnet moving inside the stator. The tubular permanent magnet EMG was designed with 12 coils wrapped around the stator. The TENG generator was based on the grating-structured free-standing TENGs with FEP/Teflon thin layers and interdigitated pairs of Cu electrodes, placed in the stator, and segmented Nylon layers, placed with the same periodicity on the slider. Using an analytical model for the dynamic response, the calculated peak OC voltage, SC current and instantaneous power density were of up to ca. 2 V, 60 mA and 210 W m^−3^ at 40 Ω for the EMG and 100 V, 20 µA and 115 W m^−3^ at 10 MΩ, for the TENG (Fig. 17a (iii, iv)). The hybrid harvester performance was also systematically explored using the irregular conditions of water waves.

A similar hybrid generator was modeled and optimized by Askari et al. (Fig. 17b) [82] to convert passing vehicle mechanical load over a speed bumper into electricity, such that the harvested energy can be used to power sensors for road traffic monitoring. It is composed of four units of sliding free-standing triboelectric PTFE/Al TENGs and four EMGs. The hybridized nanogenerator was able to deliver an output volume power density up to 20.96 and 50.81 W m^−3^ for the TENG and EMG generators, respectively, under 1 Hz, with the first predicted to become more efficient at lower frequencies of operation (< 0.5 Hz).

A hybrid energy harvester using an architecture based on the Halbach array, as depicted in Fig. 17c (i), was designed, fabricated and tested for scavenging power from low-frequency vibrations by Salauddin et al. [83, 84]. The EMG was designed with two dual Halbach arrays suspended by magnetic springs and bobbins containing two coils in such a way that the magnetic flux change through the coils was maximized. Two contact-separation TENGs were located at the ends and composed of microstructured PDMS films and Al electrodes. Under a very low-frequency range generated by human hand motion, the EMG was shown to deliver peak powers of 11.5 mW to a 32.5 Ω matched load resistance and the TENG to deliver 88 µW to a 10 MΩ load (Fig. 17c (ii, iii)).

**Fig. 18:**
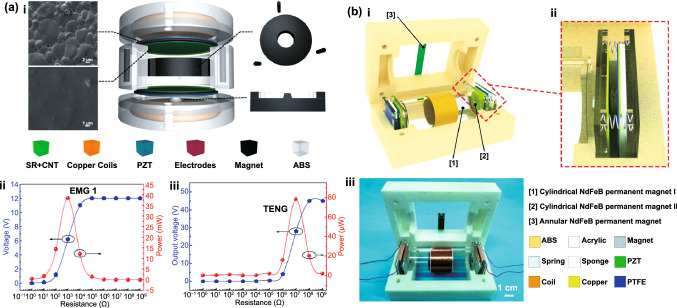
**a** (i) Triboelectric-piezoelectric-electromagnetic hybrid nanogenerator structure developed by He et al. and SEM images of the PZT and the CNT-doped silicone layers. Dependence of the output voltage and output power of the (ii) EMG and (iii) TENG on the external load resistance. Reproduced with permission from Ref. [85]. Copyright 2017, Elsevier Ltd. All rights reserved. **b** Structural design of the integrated triboelectric–electromagnetic–piezoelectric hybrid energy harvester designed by Ma et al. (i) Schematic diagram of the hybrid energy harvester. (ii) Enlarged illustration of the TENG structure. (iii) Photograph of the hybrid energy harvester. Reproduced with permission from Ref. [86]. Copyright 2019, WILEY‐VCH Verlag GmbH & Co. KGaA, Weinheim. (Color figure online)

A tri-modal hybridized triboelectric-piezoelectric (PENG)-electromagnetic nanogenerator with a magnetic levitation structure for self-powered wireless monitoring systems was developed by He et al. [85]. The structure was engineered with cylindrical symmetry with two EMGs, two piezoelectric generators and one TENG, as show in Fig. 18a (i). The EMGs were composed of a NdFeB magnet levitating in the middle of three triangle distributed magnets and two spiral-shaped coils at the edges of the container. The piezoelectric generators (PENGs) were designed with PZT circular sheets placed at the edges, such that they can be periodically impacted by the levitating magnet. The TENG consisted of a contact-mode free-standing unit between two silicone layers, with incorporated carbon nanotubes, and the levitating magnet. The maximum peak output power delivered was 38.4 mW for one EMG unit, 78.4 μW for the TENG and 122 mW for one piezoelectric unit (Fig. 18a (ii, iii)). Even though the output was non-synchronous, the combination of the three independent transduction mechanisms was still suggested to offer: a supplemental conversion with low energy losses; low mechanical fatigue or damage; operation under a small mechanical force; and a wide response range to vibration.

An integrated triboelectric-electromagnetic-piezoelectric hybrid energy harvester induced by a gravity pulled multifunction magnet was studied by Ma et al. [86] for rotational motion. As depicted in Fig. 18b, it was designed with an acrylic cylinder containing a cylindrical NdFeB permanent magnet levitating between two magnets at both ends and a coil wrapped around the middle of the cylinder. Two contact-separation mode TENGs with layers of PTFE and Cu and four supporting springs were connected to the end magnets. The piezoelectric generator consisted of a PZT beam with an annular tip magnet interacting with the moving magnet. Installed on a rotating disk, the TENG, EMG and PEG generators yielded instantaneous output powers of 712.3 μW, 30.9 mW and 6.37 μW, respectively, at 45 rpm. Charging 10 μF capacitors demonstrated the superior charging capability of the hybrid energy harvester compared with individual power generation components. Supported by a rectifier circuit and capacitor, the hybrid was able to harvest enough power for operating a thermo-hygrometer and 111 blue LEDs.

**Fig. 19:**
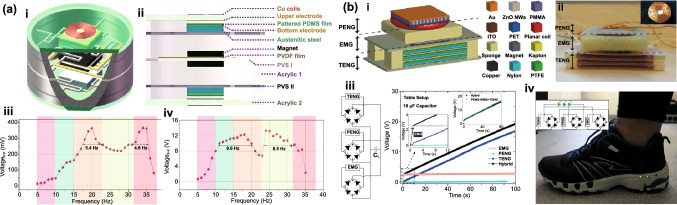
**a** (i) Schematic and (ii) cross sectional view of the low-frequency piezoelectric-electromagnetic-triboelectric hybrid broadband vibration energy harvester developed by He et al. Output peak-to-peak voltage as a function of the frequency under a 0.5 g acceleration for the (iii) EMG and (iv) TENG units. Reproduced with permission from Ref. [87]. Copyright 2017, Elsevier Ltd. All rights reserved. **b** (i) Schematic illustration and (ii) photograph of the stacked triboelectric-electromagnetic-piezoelectric hybrid nanogenerator for power-generating footwear developed by Rodrigues et al. (iii) Circuit diagram of the energy harvesting system and charging curves of a 10 μF capacitor. (iv) Photograph of the hybrid generator placed within a shoe sole and lighting up green LEDs while walking. Reproduced with permission from Ref. [88]. Copyright 2019, Elsevier Ltd. All rights reserved. (Color figure online)

Another low-frequency piezoelectric-electromagnetic-triboelectric hybrid broadband energy harvester was proposed by He et al. [87]. It was designed as a cylindrical structure with a central pair of NdFeB magnets and pickup coils, a flexible piezoelectric PVDF film and two contact-separation patterned PDMS/Cu TENGs supported by L-shaped folded PET beams (Fig. 19a (i, ii)). The device behaved as a complex multi-degree-of-freedom lumped spring-mass-damper system with three kinds of collision states and two resonance peaks for each unit (ca. at 20 Hz and 35 Hz). The low frequencies of the bending resonance modes were a consequence of the long-folded shape of the beams. Output peak-to-peak voltages of each piezoelectric, electromagnetic and triboelectric unit were observed up to 12.6 V at 20 Hz, 362.1 mV at 20 Hz and 13.3 V at 24 Hz, respectively, and the total operating bandwidths of ca. 3.5, 10.0 and 18.1 Hz (Fig. 19a (iii, iv)). A maximum output power of 41 μW at a load resistance of 800 kΩ was obtained for the piezoelectric unit, 66.5 μW at a load of 343.1 Ω for one EMG unit and 4.6 μW at a load of 1.4 MΩ for one TENG unit under 0.5 g acceleration at 20 Hz.

A stacked triboelectric-electromagnetic-piezoelectric hybrid nanogenerator for power-generating footwear was designed by Rodrigues et al. [88]. Contact-separation TENGs made up of PTFE and Nylon pairs were developed and studied with three different structures, including parallel, arcked and zigzag plates. The parallel-plate configuration generated the largest electrical outputs. Subsequently, the distance between triboelectric layers and the number of tribo-pairs in this configuration were also optimized. The final hybrid stacked structure included three parallel-plate contact-separation PTFE/nylon TENGs, a NdFeB magnet/planar coil EMG and a piezoelectric generator composed of ZnO nanowires and Au electrodes, as depicted in Fig. 19b (i, ii). The combined output of the hybrid generated superior OC voltage, SC current and power up to 75 V, 45 mA and 32 mW at ca. 100 Ω, respectively. Full diode rectifiers were implemented for the combination of the electrical outputs of the three energy harvesting units and tested for charging capacitors in a table setup and within a shoe sole during walking at constant speed (Fig. 19b (iii, iv)). The capacitors charged up to 20% more voltage in the same amount of time using the hybrid when compared to the charging voltage of the TENG alone.

**Fig. 20:**
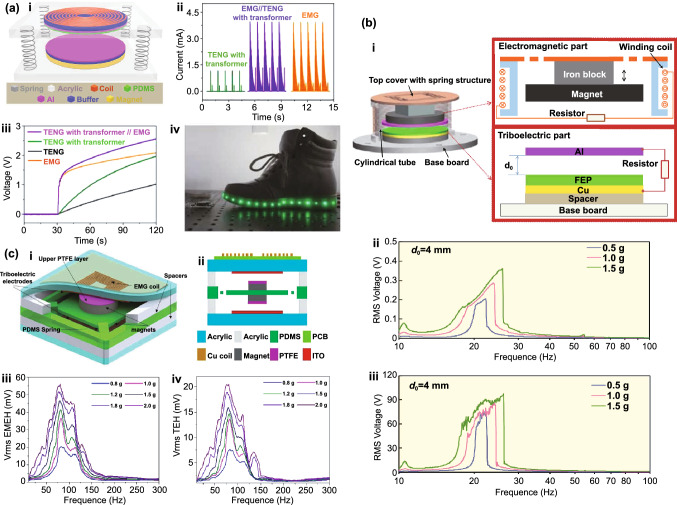
**a** (i) Schematic diagram of the hybridized nanogenerator designed by Zhang et al. (ii) SC current measured from the TENG with transformer, EMG and TENG with transformer connected in parallel with the EMG. (iii) Measured voltage across a 47 μF capacitor charged by the TENG, EMG and hybrid device (TENG with transformer in parallel with the EMG) (iv) Photograph of the hybridized generator acting as power source for on-shoe LEDs. Reproduced with permission from Ref. [89]. Copyright 2015, American Chemical Society. **b** (i) Schematic diagram of the spring hybrid generator developed by Liu et al. and cross section views of the EMG and TENG parts*.* Frequency response of the OC RMS voltage for an initial gap distance of 4 mm and for different amplitudes of acceleration of the (ii) EMG and (iii) TENG. Adapted from Ref. [90]. **c** (i) Schematic drawing of the multimodal broadband hybrid energy harvester designed by Gupta et al. (ii) Cross sectional view of the device. Frequency RMS OC voltage spectrum for the (iii) EMG and (iv) TENG at accelerations from 0.8 g to 2 g. Reproduced with permission from Ref. [91]. Copyright 2017, Creative Commons Attribution 4.0. (Color figure online)

Zhang et al. [89] developed a compact hybrid nanogenerator for scavenging biomechanical energy to sustainably power wearable electronics by human walking. As shown in Fig. 20a (i), it consisted of two square layers separated by four springs at the corners. One of the layers contains a NdFeB magnet and an Al film; the other layer comprises a spiral-shaped coil and a pyramid-patterned PDMS. Peak output powers up to 4.9 and 3.5 mW were measured for the TENG and EMG, respectively. The TENG was also connected with a transformer, adjusting its impedance to 2 kΩ, which decreased its maximum output power to 1.4 mW. Two independent rectifier bridge circuits were used for the EMG and TENG and the rectified signals connected in parallel. The hybrid device yielded a larger output average current and faster capacitor charging time than its independent constituent parts, as shown in Fig. 20a (ii, iii). Finally, the hybrid generator was integrated in a shoe to power tens of on-shoe LEDs and a smart pedometer (Fig. 20a (iv)).

A similar resonant hybrid generator providing two electrical power outputs at the same time was designed by Liu et al. [90]. As shown in Fig. 20b (i), it was formed by a cylindrical tube surrounded by a coil with a patterned folded spring copper plate on top and an attached iron block and permanent magnet. A contact-separation TENG was implemented between an Al film attached to the magnet and a FEP layer in the bottom part of the cylinder. The output performance was investigated in the time and frequency domains and at different initial gap distances and excitation accelerations. The frequency response of the RMS voltage yielded a resonance peak at ca. 23.5 Hz, getting broader for higher excitation accelerations due to nonlinear spring stiffening or for effective spring constant increasing as the magnet moved closer to the bottom base board (Fig. 20b (ii, iii)). Maximum power outputs of the EMG and TENG parts of 0.28 and 1.09 mW were measured at an acceleration of 1 g and a low resonance frequency of 23.5 Hz. The operating bandwidth could also be adjusted by changing the gap distance between the friction materials.

Gupta et al. [91] designed another multimodal broadband energy harvester using a nonlinear polymer spring (Fig. 20c (i, ii)). The EMG is an architecture with a flat coil, a pair of NdFeB magnets and a polymer spring structure, and the TENG with two contact-separating PTFE layers and ITO electrodes above and below the magnets. This device was tested with a sinusoidal mechanical vibration applied in the vertical direction with different acceleration amplitudes. As shown in Fig. 20c (iii, iv), at lower accelerations, the generators provided two voltage peaks at nearby resonant frequencies of 82 and 110 Hz, both associated with the polymer spring. At higher accelerations, a frequency broadening effect was observed due to nonlinear stiffness introduced by engagement of mechanical stoppers. The peak output RMS power of 50 μW was achieved using the EMG at an optimum load of 15 Ω, and 0.166 μW was achieved using the TENG at a load of 100 MΩ, with a wide bandwidth of operation up to 68 Hz. The hybrid device was thus able to harvest over a wide range of frequencies and accelerations. So, it was proposed its use for self-powered shock detection in automobiles and other shock sensitive applications.

**Fig. 21:**
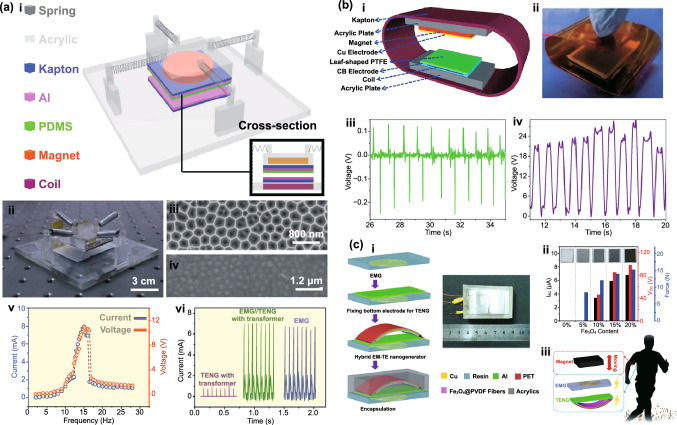
**a** (i) Schematic diagram and (ii) photograph of the hybrid nanogenerator fabricated by Quan et al. SEM images of the (iii) anodic aluminum oxide template and (iv) nanostructured PDMS film. (v) Frequency response of the output current and voltage for the EMG. (vi) Rectified output current of the TENG with transformer, EMG and hybrid nanogenerator with the EMG and TENG connected in parallel. Adapted from Ref. [92]. **b** (i) Schematic view and (ii) prototype of the hybrid EMG and TENG arc-shaped brace structure designed by Huang et al. Output OC voltage in response to a finger tapping for the (iii) EMG and (iv) TENG. Reproduced with permission from Ref. [93]. Copyright 2017, Creative Commons Attribution 3.0 Unported Licence. **c** (i) Scheme and photograph of the non-contact encapsulated E-TENG hybrid nanogenerator designed by Ren et al. (ii) Fe_3_O_4_ nanoparticles content dependence of the SC current, OC voltage and magnetic force on the PVDF/Al TENG. (iii) Schematic of the nanogenerator employed on a “self-powered coat” to harvest energy from human motions. Reproduced with permission from Ref. [94]. Copyright 2017, Elsevier Ltd. All rights reserved. (Color figure online)

Another comparable design was proposed by Quan et al. [92] featuring an acrylic platform suspended by four springs, a contact-separating TENG with PDMS and Al as triboelectric pairs and a magnet/coil EMG, as illustrated in Fig. 21a (i, ii). Nanostructures were fabricated on the surface of the PDMS film using an anodic aluminum oxide (AAO) template (Fig. 21a (iii, iv)). Under resonance conditions at 14 Hz, the TENG delivered an OC voltage of 84 V, a SC current of 43 μA and a maximum power of 1.2 mW under a load of 2 MΩ, whereas the EMG yielded 9.9 V, 7 mA and 17.4 mW under 2 kΩ (Fig. 21a (v)), respectively. Impedance matching between the TENG and EMG was achieved by using a transformer to decrease the impedance of the TENG, resulting in an enhanced rectified output current with both generators connected in parallel as shown in Fig. 21a (vi).

An arc-shaped brace structure for contact-separation energy harvesting was fabricated by Huang et al. [93] as illustrated in Fig. 21b (i, ii). It was engineered using; a common contact-separation TENG, with PTFE and Cu triboelectric pairs; and a NdFeB magnet and coil incorporated in opposite layers surrounded by an arc-shaped Kapton tape. The leaf-shaped PTFE was corona charged with an applied high voltage before measurements, thus resulting in ca. two times larger output voltages for the TENG. A random finger tap test revealed that the hybrid E-TENG ensures a high electrical performance, with respective voltage output peaks up to 0.25 and 24 V, as depicted in Fig. 21b (iii, iv). The EMG and TENG achieved an instantaneous output power density of up to 80 and 675 µW cm^−2^, respectively, for an applied acceleration of 8 m s^−2^.

Finally, a magnetic force driven non-contact encapsulated E-TENG hybrid nanogenerator for scavenging biomechanical energy was studied by Ren et al. [94]. As illustrated in Fig. 21c (i), the contact-separation TENG comprises an Al electrode and an arch-shaped membrane of PVDF fibers embedded with electrospun superparamagnetic Fe_3_O_4_ nanoparticles. The EMG was engineered using a spiral coil on the bottom side of the generator, and an external moving magnet which simultaneously excited the Fe_3_O_4_ embedded PVDF layer and induced a voltage in the coil. The magnetic force dependence of the output characteristics of the TENG was investigated as a function of the mass content of Fe_3_O_4_ nanoparticles from 0 to 20%, yielding an increase of the SC current and OC voltage of about 3–7 µA and 46–98 V, respectively (Fig. 21c (ii)). The EMG generated a peak output power of 3.4 mW under a load resistance of 200 Ω, and the TENG delivered a peak output power of 0.23 mW under 25 MΩ. A prototyped hybrid nanogenerator was embedded in a “self-powered coat” to harvest energy from human arm motions, as illustrated in Fig. 21c (iii). It was observed the ability of this hybrid E-TENG to charge energy storage devices and sustainably power some portable electronics, such as a digital watch and a thermo-hygrometer.

### Sliding Generators

**Fig. 22:**
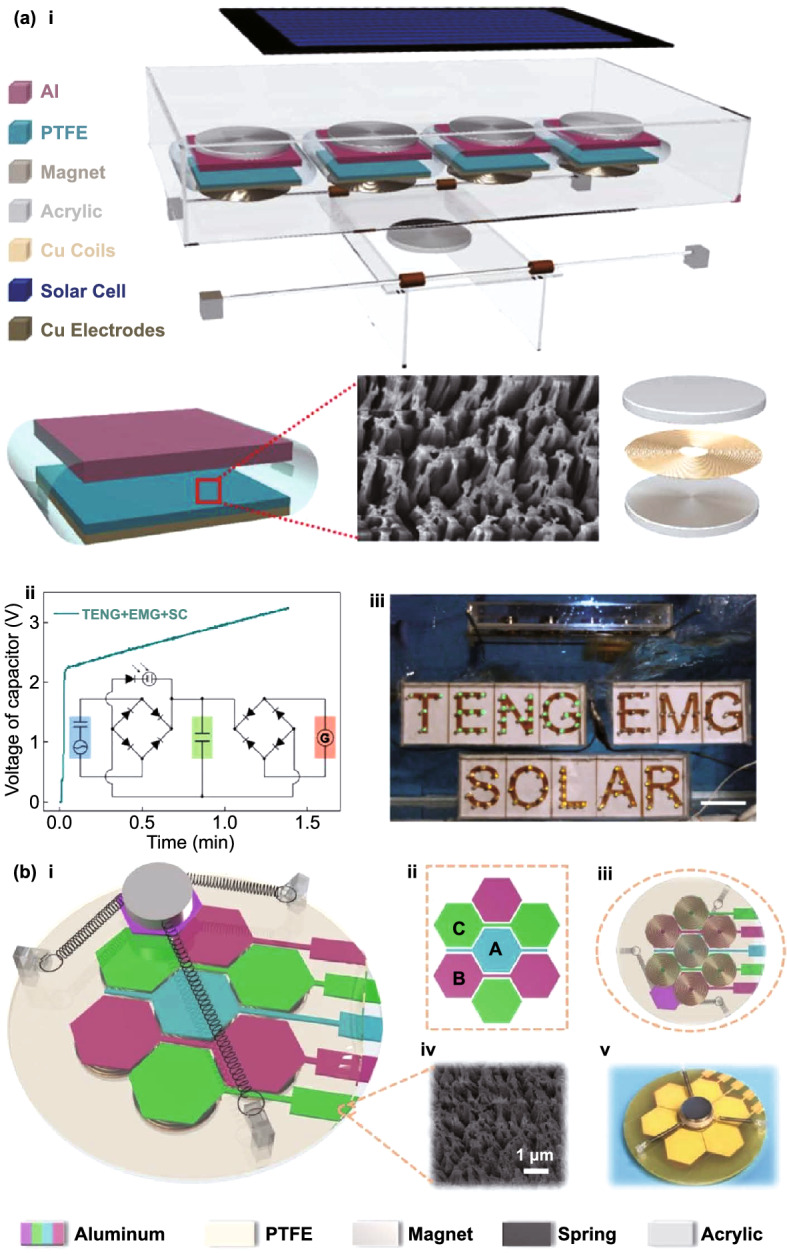
**a** (i) Structure design of the multifunctional hybrid power unit for harvesting blue energy fabricated by Shao et al., composed of four repeated contact-separation TENGs, corresponding free-standing sliding-mode EMGs and a waterproof silicon-based solar cell. (ii) Measured voltages of a 33 μF commercial capacitor charged by the hybrid power unit. (iii) Demonstrations of the hybrid power unit as a practical power source. Reproduced with permission from Ref. [95]. Copyright 2017, Elsevier Ltd. All rights reserved. **b** Structural design of the hybridized ocean wave nanogenerator developed by Feng et al. (i) Schematic diagram. (ii) Structure of the electrodes of the TENG units. (iii) Copper coils of the EMG units in the bottom. (iv) SEM image of the PTFE surfaces. (v) Photograph of the nanogenerator. Reproduced with permission from Ref. [96]. Copyright 2018, Elsevier Ltd. All rights reserved. (Color figure online)

Five hybrid E-TENG technologies were already designed based on sliding architectures. A multifunctional power unit by hybridizing contact-separation TENG nanogenerators, EMG generators and solar cells for harvesting blue energy was proposed by Shao et al. [95]. As depicted in Fig. 22a (i), it consisted of a fully enclosed acrylic box containing four nanostructured PTFE/Al contact-separation mode TENGs placed in a row, corresponding free-standing sliding-mode EMGs, each with a top magnet and a bottom disk-type copper coil, and commercial waterproof silicon-based solar cells placed on top. When harvesting ocean wave kinetic energy, a bottom magnet, attached to a sliding piece of acrylic sheet, moved back and forth, making the magnets inside the box shake upward or downward. Under a simulated ocean wave of 2 Hz, the TENGs produced OC voltages, SC currents and average powers up to 142 V, 23.3 μA and 31.5 μW, respectively, and the EMGs provided 0.66 V, 2.14 mA and 66.9 μW. The rectified hybrid power unit has been demonstrated as a practical power source to drive LEDs directly or charge commercial supercapacitors under all weather conditions (Fig. 22a (ii, iii)). The TENGs offer high output at low frequencies (< 0.5 Hz) and the EMGs provide high output at higher frequencies of the waves.

An hybridized nanogenerator based on honeycomb-like three electrodes for efficient ocean wave energy harvesting was designed by Feng et al. [96]. As shown in Fig. 22b, its main component was a magnet suspended by three springs fixed on the edge of an acrylic board. This board contained honeycomb-like electrodes (seven hexagonal Al films divided in three groups, named ‘A’, ‘B’ and ‘C’), covered with a nanostructured PTFE film, and copper coils fixed on the back of these electrodes. The TENGs were housed between the PTFE and Al electrode on the magnet, such that they could work in both in-plane sliding-mode and vertical contact-separation mode. With a displacement of 3.5 cm at a fixed working frequency of 4 Hz, the TENG yielded an OC voltage of 550 V, SC current of 1.25 µA and maximum output energy of 21.7 µJ, while the EMG yielded 3 V and 4.65 mA, respectively, and an average power of 8.23 µW. This structure was designed to harvest wave energy in multi-angle range for random waves. In addition, by adjusting the weight of the magnet to achieve different resonance parameters, the generator could reach a resonant state and corresponding more efficient harvesting of ocean energy.

**Fig. 23:**
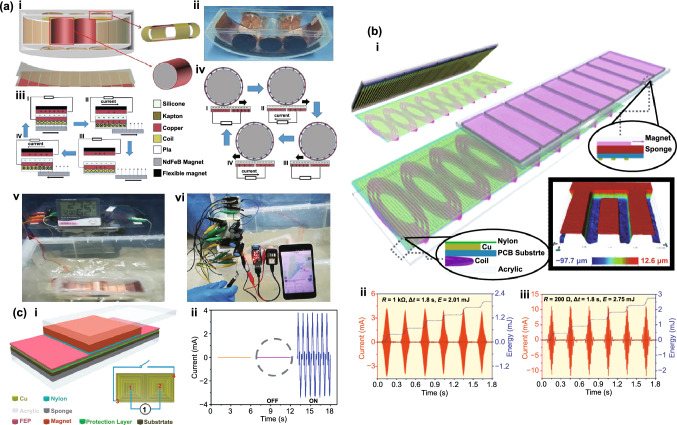
**a** Structural design of the ship-shaped hybrid nanogenerator for blue energy harvesting developed by Wang et al. (i) Schematic diagram of the device with a free-standing TENG on the bottom and contact-separation TENGs and EMGs on both sides. (ii) Photograph of the generator. Schematics diagrams of the working mechanisms of the (iii) EMG and contact-mode TENG and (iv) sliding-mode TENG. (v) Application of the generator for powering a humidity and temperature sensor. (vi) Application demonstration of the generator driving a general packet radio service. Reproduced with permission from Ref. [97]. Copyright 2018, Published by Elsevier Ltd. **b** (i) Structural design of the linear-grating hybridized nanogenerator developed by Zhang et al. and detail of the interdigitated ultramicroelectrode array. Output current and energy for the (ii) TENG and (iii) EMG after using transformers under an acceleration of 20 m s^−2^. Adapted from Ref. [98]. **c** (i) Schematic diagram of the sliding shared-electrode-based hybridized generator designed by Quan et al. (ii) Measured output current signals of the hybridized nanogenerator after turning a switch. Reproduced with permission from Ref. [99]. Copyright 2016, American Chemical Society. (Color figure online)

A fully packaged ship-shaped hybrid nanogenerator for blue energy harvesting toward seawater self-desalination and self-powered positioning was developed by Wang et al. [97]. It consisted of a ship-shaped box containing: a free-standing lateral-sliding TENG on the bottom part; six contact-separation mode TENGs; and EMGs arranged on the two sides (Fig. 23a (i–iv)). The free-standing TENG was incorporated between a grating of Cu-metal electrodes, located on the bottom stator covered by silicone, and up to three copper-wrapped round tubes, employed as rollers with two round cake magnets at both ends (Fig. 23a (iv)). The contact-separation TENGs and EMGs were formed by a stack of a flexible magnet and Cu attached to a stack of silicone, Cu and coils (Fig. 23a (iii)). This rolling design should effectively attain resonance with water waves due to the low natural frequency of the roller itself, as well as due to low frictional resistance. Under a simulate water wave movement, the largest instantaneous output power of the free-standing TENG reached 165 µW at a load resistance of 20 MΩ, while the contact-separation TENG and EMG harvested 850 µW at 20 MΩ and 9 mW at 100 Ω, respectively. With commercial transformers employed to decrease the impedance of the TENG, the hybrid generator was shown to provide a higher capacitor charging voltage and faster charging speed than by using the individual TENGs and EMG. When driven by low-frequency water waves, this hybrid generator was able to effectively power a desalinating seawater device and a digital temperature-humidity meter, as well as to drive a radio-frequency emitter to form a self-powered wireless positioning system (Fig. 23a (v, vi)).

Zhang et al. [98] reported a linear-grating hybridized nanogenerator for scavenging the mechanical energy generated from sliding motions to sustainably power portable electronics. Its structural design consisted of a slider and a stator with 66-segment sliding free-standing TENG and a 9-segment EMG. The TENG was designed with a layer of grated Cu ultramicro-strips and a nylon film; the EMG is composed by nine rectangular alternate magnets and coils (Fig. 23b (i)). At a sliding acceleration of 20 m s^−2^, the hybridized nanogenerator delivered maximum powers of 102.8 mW for the TENG at 0.4 MΩ and 103.3 mW for the EMG at 6 kΩ (Fig. 23b (ii, iii)). A power management circuit comprising transformers, rectifiers, voltage regulators and capacitors was also engineered to ensure their combined operation, which allowed to provide a sustainably powering of a digital clock and a thermometer only through sliding by hand.

A sliding shared-electrode-based hybridized E-TENG nanogenerator was designed by Quan et al. [99] to simultaneously harvest mechanical energy from one motion. Two planar Cu coils were employed as electrodes for both the TENG and EMG with a mechanical switch. As shown in Fig. 23c (i), the hybrid generator was composed of a fixed layer with a FEP film, protection layer, two coils, a sponge film and an acrylic substrate, as well as a moving part with a nylon film, magnet and acrylic substrate. The hybridized nanogenerator was capable to deliver 3.8 mA output current and 245 V output voltage when the switch in the device circuit was turned on and off, respectively (Fig. 23c (ii)). A self-powered acceleration sensor was demonstrated having detection sensitivities of about 143.2 V (m/s^2^)^−1^ for the TENG and 291.7 μA (m/s^2^)^−1^ for the EMG.

### Cantilever Generators

**Fig. 24:**
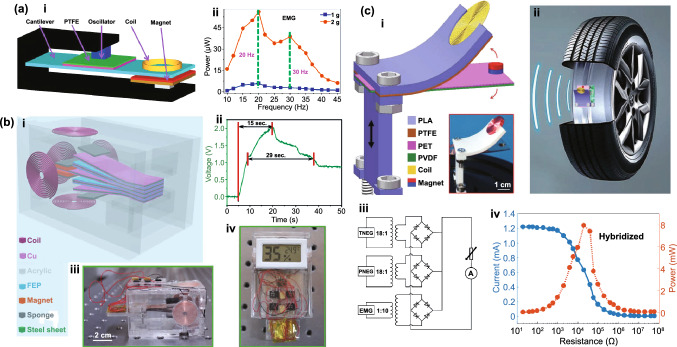
**a** (i) 3D schematic view of the hybrid EMG and TENG multi-impact energy harvester designed by Zhu et al. (ii) Measurement power as a function of the frequency and two different excitation acceleration amplitudes for the EMG. Reproduced with permission from Ref. [100]. Copyright 2017, Creative Common CC BY license. **b** (i) 3D schematic diagram of the hybrid nanogenerator devised by Quan et al. (ii) Measured voltage of a 200 μF capacitor connected to the hybrid nanogenerator and a temperature–humidity sensor. (iii) Photograph of the fabricated nanogenerator. (iv) Photograph of the self-powered temperature–humidity sensor system. Adapted from Ref. [101]. **c** (i) Structural design of the cantilever EMG–TENG–PENG hybridized nanogenerator reported by Du et al. (ii) Design of the self-powered tire pressure monitoring system (iii) Circuit diagram of the energy harvesting system including a TENG, PENG and EMG with transformers and rectifiers. (iv) Dependence of the output current and power of the hybridized nanogenerator on the external loading resistance. Reproduced with permission from Ref. [102]. Copyright 2018, WILEY‐VCH Verlag GmbH & Co. KGaA, Weinheim. (Color figure online)

The cantilever architecture was also used by three hybrid E-TENG technologies, as follows. A resonant cantilever-based hybrid generator, using a nonlinear multi-impact approach for broad-bandwidth-frequency (10–45 Hz) energy harvesting, was designed by Zhu et al. [100]. The harvester includes a long acrylic cantilever beam with a cylindrical coil located at its free tip and a magnet fixed at the base of the cantilever; a free-standing TENG consisting of a PTFE film located at the middle plane of the cantilever and a Cu stopper in the base (Fig. 24a (i)). Due to the nonlinearity of the impact and the constraint space, the dynamic response of the cantilever presented an impact driven frequency up-conversion and a much broader bandwidth with two resonant peaks at 20 Hz and 30 Hz (Fig. 24a (ii)). Maximum average power densities of 38.1 µW m^−2^ were obtained for the EMG and 30 µW m^−2^ for the TENG.

A more complex fully enclosed resonant cantilever hybrid generator consisting of five EMGs and four TENGs was devised by Quan et al. [101]. The generator was composed of five steel cantilever sheets with four integrated FEP/Cu contact-separation TENGs, five coils placed on the surfaces of an acrylic box, and two magnets fixed at the free tip of the middle steel sheet, as depicted in Fig. 24b (i, iii). Under a vibration frequency of 15.5 Hz, one TENG delivered powers up to 0.13 mW under a load resistance of about 0.8 MΩ, while one EMG delivered up to 0.08 mW under a load of 3 kΩ. This hybrid E-TENG generator (rectified EMGs connected in series and rectified TENGs in parallel) was able to power a temperature–humidity sensor for 29 s after scavenging the vibrational energy produced by human hands and charging a 200 μF storing capacitor from 0 to 2 V in 15 s (Fig. 24b (ii, iv)).

A cantilever hybridized nanogenerator based on E-TENG–PENG for an highly efficient conversion of vibrational mechanical energy into electricity was reported by Du et al. [102]. As illustrated in Fig. 24c (i), the hybrid was designed by including: a PET–PVDF unimorph cantilever with a cylindrical magnet as a tip load and a coil suspended on a PLA-PTFE cambered shell; a contact-separation TENG between the PET and the Al coated nanoparticle-based PTFE; and a piezoelectric PVDF layer. Integration with transformers and rectifiers was carried out to achieve the impedance match among the TENG, PENG and EMG (Fig. 24c (iii)). Under the device resonance frequency of 23 Hz, the TENG delivered output powers up to 1.2 mW at 0.4 MΩ, the EMG up to 7.4 mW at 40 kΩ and the PENG up to 1.1 mW at 40 kΩ. The hybridized nanogenerator provided up to 8 mW at 20 kΩ (Fig. 24c (iv)). Through a power management circuit integrated into the hybridized nanogenerator, a continuous DC output was achieved, providing sufficient power to drive a radio­frequency wireless tire­pressure monitoring system (Fig. 24c (ii)), as well as other conventional electronics.

### Flexible Blade Generators

**Fig. 25:**
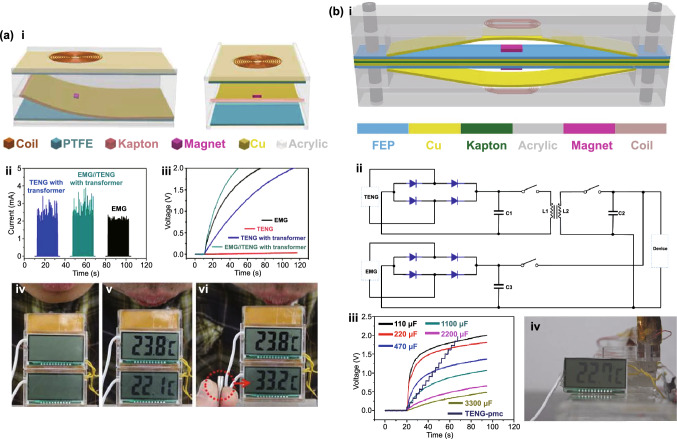
**a** (i) Schematic diagram of the hybridized air-flow E-TENG nanogenerator designed by Wang et al. (ii) Rectified output current signals of the individual TENG unit, EMG unit and both units connected in parallel. (iii) Measured charging curves of a 3300 μF capacitor by using the different devices. (iv–vi) Scavenging of human mouth blown air-flow energy to sustain two temperature sensors. Reproduced with permission from Ref. [48]. Copyright 2015, American Chemical Society. **b** (i) Schematic diagram of the hybridized nanogenerator fabricated by Wang et al. (ii) Designed power management impedance matching switching circuit with multiple capacitors for the hybridized nanogenerator. (iii) Measured charging curves of different capacitors by the EMG and 110 μF by the TENG with power management circuit (PMC). (iv) Photograph of a temperature sensor being powered by the generator. Reproduced with permission from Ref. [103]. Copyright 2016, Elsevier Ltd. All rights reserved. (Color figure online)

Two hybrid E-TENG technologies using flexible architectures were also investigated. An hybridized nanogenerator with two flexible TENGs and two EMGs was developed by Wang et al. [48] to harvest air-flow energy. As depicted in Fig. 25a (i), it consisted of an acrylic tube integrated with a TENG, composed of two fixed PTFE films and a free vibrating Kapton film, and a EMG, composed of two planar coils and a magnet fixed on the Kapton film. Maximum output powers of 3.5 mW were obtained for one TENG at a load of 3 MΩ and 1.8 mW for one EMG at 2 kΩ under an air-flow speed of ca. 18 m/s. Four hybridized nanogenerators with the TENGs connected to transformers were able to charge a Li-ion battery to sustainably power four temperature sensors with human blown air, as well as to drive a wireless temperature sensor with a nanogenerator charged Li-ion battery (Fig. 25a (iv–vi)).

Wang et al. [103] also reported a nanogenerator for scavenging wind energy power, as well as a power management circuit for effective energy storage. As depicted in Fig. 25b (i), the nanogenerator included two contact-separation TENGs between a central flexible FEP layer, fixed at two end points, and Cu electrodes; and two EMGs composed by two permanent magnets, placed in the central FEP layer, and coils anchored on the structure frame. The TENGs delivered a peak output power of 1.7 mW for a resistance of 10 MΩ, while the EMGs yielded about 2.5 mW for a load of 1 kΩ under a wind speed of about 18 m s^−1^. To solve the issue of high impedance of the TENG, as well as to carry out an efficient energy storage using a capacitor, a power management circuit was designed including two bridge rectifiers, three capacitors, a coupled inductor and an on/off switch (Fig. 25b (ii)). The charging rate of a capacitor has been optimized using the TENG by changing the inductance and the switching on/off time for the charge transference between the smaller and larger capacitors (Fig. 25b (iii)). The hybridized E-TENG nanogenerator with management circuit thus showcased the ability to power a temperature sensor for a much longer time (Fig. 25b (iv)).

### Multidimensional Generators

**Fig. 26:**
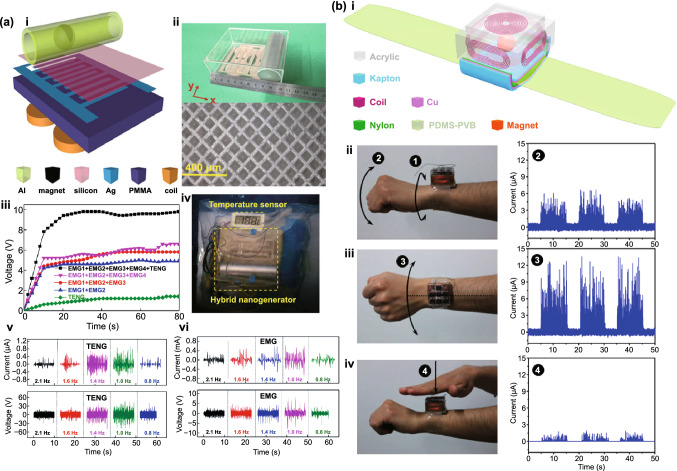
**a** (i) Schematic diagram of the two-dimensional wave energy hybrid harvester developed by Hao et al. (ii) Picture of the hybrid nanogenerator and SEM image of the silicone thin film with pyramidal structures. (iii) Charging curves of a 10 μF capacitor by the nanogenerator at a frequency of 1.5 Hz. (iv) Thermometer powered by the hybrid nanogenerator under the conditions of water wave vibration. SC current and OC voltage of the (v) TENG and (vi) EMG under different water wave frequency. Reproduced with permission from Ref. [104]. Copyright 2019, Elsevier Ltd. All rights reserved. **b** (i) Schematic diagram of the three-dimensional magnetic ball-based bracelet hybridized nanogenerator designed by Quan et al. (ii) Photograph of three different wrist motion types and corresponding output current signals. Reproduced with permission from Ref. [105]. Copyright 2015, American Chemical Society. (Color figure online)

Five multidimensional architectures for hybrid E-TENG technologies were already proposed. Hao et al. [104] developed a box-like enclosed hybrid nanogenerator for two-dimensional (2D) low-frequency wave energy harvesting. As shown in Fig. 26a (i, ii), the free-standing TENG was composed of an Al-coated hollow acrylic cylinder sliding over a pyramid-patterned silicone film with interdigitated electrodes. The EMG was formed by a NdFeB magnet ball moving inside the cylinder and four copper coils embedded in the lower part of the box. Under an excitation of 1.8 Hz, the measured output peak SC current and OC voltage for the TENG were of up to 1.2 μA and 75 V, respectively, and up to 1 mA and 9 V for the EMG. The TENG provided an instantaneous maximum output power up to 0.08 mW at a load of 100 MΩ, while the power of the EMG reached 14.9 mW at a load of 1 kΩ. The different units were connected to full-wave rectifying bridges and used to charge a 10 μF capacitor. The E-TENG achieved the highest charging speed (Fig. 26a (iii)). Besides, it was able to continuously drive a temperature sensor (Fig. 26a (iv)), lighting up 60 LEDs, and to power a wireless acoustic sensing system while working in a water wave environment. The ability of the hybrid nanogenerator for a better collection of the wave energy in 2D space was also studied under different wave frequencies and amplitudes, and with different tilt angles (Fig. 26a (v, vi)).

Quan et al. [105] designed a brace nanogenerator with one TENG and six EMG units triggered by wrist motions to charge a Li-ion battery for a self-powered electronic watch. Figure 26b (i) illustrates the schematic diagram of the device. The EMG is composed by a magnetic ball placed in an acrylic box with flat coils on each side. A contact-separation TENG was also included at the bottom of the box made up of a nylon film and a PVB nanowire/PDMS composite film. The TENG delivered output power up to 0.1 mW under a loading resistance of 6 MΩ; the EMG provided about 2.8 mW under a load of 700 Ω. The device was also tested for multiple types of arm motion yielding current peaks of up to ca. 13 mA (Fig. 26b (ii–iv)).

**Fig. 27:**
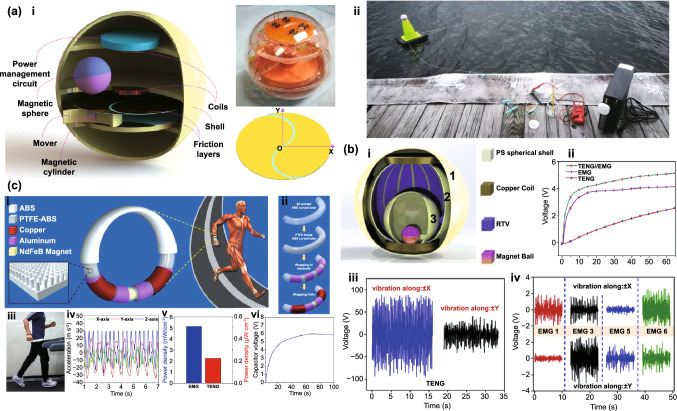
**a** (i) Schematic diagram, photograph and friction electrodes of the hybridized water wave energy harvester designed by Wu et al. (ii) Photograph of the self-powered wireless water temperature alarm system with the generator placed in a buoy being tested in a lake. Reproduced with permission from Ref. [106]. Copyright 2019, American Chemical Society. **b** (i) 3D full-space E-TENG hybrid nanogenerator structure designed by He et al. (ii) Charging curves of a 10 μF capacitor by the TENG, EMG and hybrid modules. Output OC voltage with different excitation directions (along a *X* or *Y* axis with the sphere rotating between silicon layers or along a single layer, respectively) for the (iii) TENG and (iv) EMGs. Reproduced with permission from Ref. [38]. Copyright 2020, Elsevier Ltd. **c** (i) Wearable hybridized E-TENG nanogenerator for harvesting biomechanical energy from arm swinging motion designed by Maharjan et al. (ii) Stepwise assembly process of the harvester. (iii) Photograph showing fast walking activity and (iv) corresponding 3-axis waveform accelerations. (v) Power density delivered by the EMG and TENG and (vi) voltage charge curve of a 470 μF capacitor by the hybrid generator, connected to voltage multiplier circuits and rectifiers, associated with the fast walking. Reproduced with permission from Ref. [107]. Copyright 2018, Elsevier Ltd. (Color figure online)

Wu et al. [106] designed an hybrid energy harvester based on a magnetic sphere capable of harvesting blue water wave energy from any orientation. This generator was engineered with two TENGs and a EMG, a power management circuit and a spherical acrylic shell, as depicted in Fig. 27a (i). The lateral-sliding TENG modules were designed by placing a Cu coated magnetic cylinder mover between two PTFE friction layers; the EMG module was designed by placing a NdFeB sphere, magnetically coupled to the magnetic cylinder, between two self-adhesive coils. At 30 rpm and with a 135° direction orientation, the output of the parallel TENG module reached a maximum voltage value of ca. 60 V and a current of ca. 1.2 µA, while the series EMG module reached 1.7 V and 1 mA, respectively. When the EMG and TENG units were connected to a rectifying circuit, and then connected in series, it was able to power a water temperature alarm by incorporating it within a buoy flotation device in a lake (Fig. 27a (ii)).

A 3D hybrid nanogenerator based on a magnetic ball and spherical shells to transform 3D full-space mechanical energy into electrical energy from multiple methods was also developed by He et al. [38]. As shown in Fig. 27b (i), the structure was formed by three concentric Polystyrene (PS) spherical shells with the EMG module made up of a central magnet ball and six spiral copper coils in between the two outer plastic shells. The grating free-standing TENG module was formed by the inner shell and multiple pyramid-patterned vulcanized silicone rubber (RTV) friction layers on the inner surface of the second shell. The performance of the TENG and EMGs was studied as a function of the direction of external vibration and excitation frequency. The TENG was able to harvest up to 77 V of OC voltage and up to 0.7 µA of SC current peaks for excitation direction along an *X* axis (with the sphere rotating between silicon layers) and up to 40 V and 0.3 µA for an excitation along an *Y* axis (with the sphere moving along a single silicon layer) (Fig. 27b (iii)). The electrical output for a single coil reached 2.4 V and 1 mA under an excitation frequency of 2 Hz (Fig. 27b (iv)). Overall, a maximum output power of 18 µW at a load of 200 MΩ was obtained for the TENG and 640 µW at 1 kΩ for the EMG. The seven different electricity modules connected in parallel by a rectifier circuit were shown to charge a 10 µF capacitor faster and up to higher voltages than its components (Fig. 27b (ii)). Besides, it was able to light up 200 commercial LEDs and continuously power humidity and temperature sensors.

A curve-shaped wrist-wearable lightweight low-frequency nanogenerator was manufactured by Maharjan et al. [107]. As shown in Fig. 27c (i, ii), it was designed as a hollow curved tube, in which the EMG is composed by a NdFeB magnetic ball and two wrapped copper coils, and the free-standing TENG formed by the PTFE coated tube walls, the metal ball and Al electrodes. The hybrid generator’s performance was tested under different 3D biomechanical motion conditions (Fig. 27c (iii–vi)). The EMG delivered a peak power density of 5.15 mW cm^−3^ at an optimum load resistance of 49 Ω, while the TENG delivered a peak power density of 0.223 µW cm^−3^ at an optimum load resistance of 13 MΩ. It was also demonstrated its ability to drive a commercially available electric wrist-watch and a self-powered heart-rate sensor.

### Magnetoelectric Generators

**Fig. 28:**
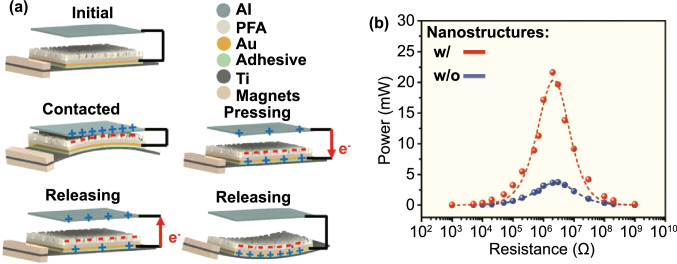
**a** Schematic of the magneto-mechano-triboelectric generator designed by Lim et al. **b** Peak power measured at the resonance frequency as a function of the load resistance, with and without nanostructures in the PFA film. Adapted from Ref. [108]. (Color figure online)

Only a hybrid magneto-mechano-triboelectric technology was developed up to date. It was proposed by Lim et al. [108] to convert an alternating current’s (50/60 Hz) small magnetic field (< 1 mT) into electrical energy for powering a wireless indoor positioning system. It was projected as a long cantilever with: a tip proof NdFeB magnet; a contact-separation TENG formed by a nanostructured PFA film, located on top of the cantilever; and an Al foil located on the overhead area of the PFA surface (Fig. 28a). The device generated an OC peak-to-peak voltage and a SC current of 708 V and 277 mA, respectively, as well as a maximum peak power of 21.8 mW under a magnetic field of 7 Oe at a resonance frequency of 143.2 Hz (Fig. 28b).

## Comparison of Various E-TENG’s Designs

The most relevant analyses of E-TENGs are summarized in Table 1. It includes: (i) the TENG materials and operation mode; (ii) the E-TENG dimensions; (iii) the energy source and driven excitation; (iv) the TENG performance; (v) the EMG performance; and (vi) the E-TENG performance. The reported largest single generator was designed by Chen et al. [74] with ca. 1300 cm^3^; the smallest generator was developed by Huang et al. [93] with ca. 5 cm^3^. The main mechanical energy sources were human motion, machine vibration, water waves, water/wind flows and rotating tires. Generators were mechanically driven by the excitations mostly in the 0.5–2 g and 1–25 Hz range. TENGs were able to provide OC voltages in the range between 4.5 and 708 V, SC currents between 42 nA and 4.2 mA and maximum instantaneous power of 217.8 mW. EMGs were able to provide OC voltages in the range between 0.1 and 55.7 V, SC currents between 0.5 and 115.88 mA and maximum instantaneous power of 265 mW. Regarding the E-TENG hybrid generators, OC voltages in the range between 110 mV and 75 V, SC currents between 7 µA and 60 mA and maximum instantaneous power of 144.1 mW were achieved. Roughly speaking, linear generators yielded the largest average power densities of ca. 0.5 mW cm^−3^, followed by rotational generators with ca. 0.3 mW cm^−3^ and multidimensional generators with ca. 0.1 mW cm^−3^. This is possibly the case due to a poor optimization of the multidimensional design in relation to the other two, because of an infrequent excitation of the multiple TENG units and a large average distance between magnets and coils.

Rotational E-TENGs are particularly well suited for scavenging energy from water and wind flows together with rotor blades converting the linear motion into rotational motion. Cylindrical structures with multiple generator units along the radial direction can be easily fabricated to yield a frequency multiplication effect, which is useful for low input frequencies. Free-standing TENGs with enhanced output current can be straightforwardly integrated and the friction can be minimized using various strategies. New approaches could still be found to maximize the electromechanical coupling depending on the shape and relative positioning between magnets and coils. Optimal turbine design for the specific fluid flow conditions could be explored by using computational fluid dynamics.

Pendulum generators are associated with an efficient conversion between gravitational potential energy and kinetic energy, thus making them ideal for multidirectional (along angular orientations) random and very low-frequency inputs, such as those of water waves. Matching the resonance frequency with the external source and employing coupled pendulums could be sought after. Since the output power is *ca.* proportional to the powers of the relative velocity between parts of the generators, strategies employing a gradual build-up of gravitational potential energy up to a threshold followed by a sudden release could be explored.

Generators with a linear architecture can be used to harvest energy from a wide array of vibration sources, including biomechanical, passing vehicles, working machinery and blue energy. They have a simple design and fabrication process, which makes them relatively easy to miniaturize, and their great robustness is suitable for operation under intensive cyclic motions and intermittent impacts. Resonant systems are easily assembled and can also be integrated with piezoelectric generators. Wider frequency bandwidths of operation could still be pursued by employing self-adaptive mechanisms, nonlinear restoring forces or multiple moving components. Linear generators could be integrated with both contact-separation and sliding free-standing TENGs. The lower frequencies and amplitudes of operation could be decreased by employing low-friction materials, interface liquid lubrication or operation under partial vacuum.

Sliding generators are similar to linear generators, although they require larger cross area-to-length ratios and are mostly sensitive to lateral-sliding or shear-like motions. These structures can easily combine resonant behavior and, like rotational generators, frequency multiplication. Possibly larger relative displacements between moving parts can be obtained if one of the components was to be anchored to an inert referential, such as the sea floor.

Cantilever-shaped generators are able to provide low-frequency sharp resonance peaks, as well as large strains and stresses along the length of the structure. However, due to their characteristic bending deformation, their integration with traditional TENGs and EMGs is difficult to carry out, since the relative positioning between the moving and static parts is hard to optimize. Piezoelectric generators are ideal to be combined with these structures.

Flexible blade generators comprise thin flexible sheets, which are sensitive to weak air currents, and thus well fitted toward harvesting air-flow energy. Like the cantilever structures, they are difficult to integrate with bulky EMGs. Flexible bladed TENGs and PENGs can be miniaturized and combined in vast arrays of devices.

Multidimensional generators are able to extract power from all kinds of movement and vibration sources. However, they take up relatively large volumes, the relative positioning between their moving parts are hard to optimize and the outputs from multiple generation units are difficult to synchronize. Single magnetic spheres with changing magnetic vector orientations should also be subject to the effect of magnetic torques, thus tending to decrease the magnetic flux change across the coils. Integration of multiple magnetically coupled spheres in different stages can be useful in this regard.

Magnetoelectric generators, which are able to convert both mechanical energy and energy from magnetic fields into electrical energy, can also be useful in the near future to extract energy from unused stray magnetic fields, such as those from working machinery and geomagnetic anomalies.

In general, output currents from TENGs and voltages and currents from EMGs were shown to increase linearly with the input frequencies and amplitudes of excitation. Output voltages from TENGs were observed to remain *ca.* stable with the same parameters, thus resulting in superior power outputs under conditions of low input frequencies and amplitudes. The hybridization of both generators and their integration with rectification, and energy storage systems provide a combination of faster charging times, determined by the higher output currents of the EMGs, and the larger final voltages ensured by the higher output voltages of TENGs.

The fundamental working principle of the motion-driven electrical generators is based on the introduction of electromechanical damping of movement and conversion of part of the input kinetic energy into electrical energy. Therefore, when they are integrated together in hybrid devices, both EMGs and TENGs are expected to somewhat compete for extracting power. Nevertheless, since most reported E-TENGs typically feature energy conversion efficiencies lower than *ca*. 30%, the maximum output powers across a resistive load (not necessarily optimal) tend to be a simple mathematical sum [63, 64, 76]. These performances are expected unless the internal mechanical damping factor introduced by each generator is comparatively large [164]. On the other hand, most tested hybrid devices yield unimpressive results when compared to the individual generators, most likely due to the problem of the huge impedance mismatch and the simple direct charging methods with high loss transformers that have been used. Thus, large improvements still have to be carried out in the scope of power management and storage (as discussed in the next section). In any case, the main purpose of E-TENG generators is to enhance the energy conversion efficiency over a wider range of input powers, amplitudes and frequencies, and not necessarily the output power for the same input. Different volume scaling behavior for each kind of generator must also be taken into account when integrating both devices.

## Concluding Remarks and Future Prospects

This review deeply and systematically explores the most relevant breakthroughs already carried out in the scope of hybrid triboelectric-electromagnetic nanogenerators driven by mechanical energy harvesting. The theoretical transduction mechanism models developed for both TENGs and EMGs were exposed, as well as reported structural design prototypes and corresponding electromechanical characteristics and applications. Hybridized E-TENGs can offer a more efficient vibration energy conversion by taking advantage of both of their desirable complementary high voltage and high current characteristics and wider operating bandwidths. The TENGs are effectively able to harvest electric energy from low-frequency (< 1 Hz) and low amplitude (< 1 mm) kinetic energy, providing large output voltages. The EMGs are effective technologies to harvest at high frequencies and amplitudes of excitation, providing high output currents. E-TENGs can also be connected in series or in parallel, or used independently, to output high voltages or currents to fulfill customized requirements and suitability for particular applications. Thus, by yielding peak output powers higher than 100 mW, they might find use as micro/nanopower sources or in self-powered sensors by scavenging general forms of vibration energy, wheel rotation energy, biomechanical energy, blue energy, wind energy, thermal energy and more. Therefore, E-TENG generators hold potential to power captivating technologies, such as those related to the internet of things, wireless sensor networks, portable electronics, implantable biomedical devices, etc. Despite the observed impressive progress, some important issues remain to be addressed:

(i) Energy conversion efficiency—The energy scavenging capabilities of TENGs are still not sufficient to continuously power most conventional electronic devices. Since its output power increases with the square of the triboelectric surface charge density, enhancing this value is mandatory. At least five different strategies have been explored in this regard: modification in material composition, improvement in effective contact area, surface charge pumping, ionized-air injection and control of environmental conditions. The composition of the triboelectric materials can be modified through chemical surface functionalization [182], by changing the functional groups on the surface or bulk composition manipulation [183], by using high dielectric constant materials as fillers, such that the charge-attracting or charge trapping capability of the materials can be enhanced. The effective contact area can be improved by introducing nano/microstructures onto the triboelectric materials, such as nanowires and pyramid/cube-like arrays [137, 184] or by using fluid–solid interfaces [185]. The charge density of a triboelectric nanogenerator can be pumped up by a charge-shuttling technique [186] or enhanced by ionized-air injection [187]. Charge accumulation strategy was successfully employed to boost the output voltage providing sustainable OC voltages over 20 kV in a TENG with rotating tribo-electrodes and transporting electrodes [188]. Controlling environmental conditions such as temperature and air pressure to increase surface charge density and dielectric breakdown voltage is another valid strategy [189].
(ii) Power management—Coupling the output powers from the TENG and EMG units is a tremendous issue due to the huge impedance mismatch between them and between TENGs and storage devices. This is also complicated by the dependence of the equivalent internal impedance of TENGs and resonant EMGs with the excitation frequency. A better understanding of the combined energy harvesting efficiency in hybrid systems is required. It is also relevant the conversion of irregular AC electrical outputs into stable DC input power to supply electronic systems. The use of conventional rectifiers and transformers introduces large energy losses so that advanced power management methods to achieve high energy harvesting efficiency are critical [190]. Impedance matching has already been achieved with TENGs by designed complex power converters, employing multiple temporary storage capacitors, coupled inductors and switches controlled by logic units [103, 191]. In such systems, a first stage with a small temporary capacitor (with large equivalent impedance) is charged through a bridge rectifier from 0 V. Once the voltage reaches an optimal level after the impedance match condition is achieved, the energy stored in the temporary capacitor is transferred to another stage with a storage unit (e.g., a large capacitor) through coupled inductors. Thus, such an optimized charging cycle can lead to a maximization of the charging efficiency up to *ca.* 75% [190]. Another strategy makes use of a group of capacitors that are periodically charged in a series connection and discharged to a load in a parallel connection [192]. Energy transfer maximization has also been explored through a rationally modulated charging cycle controlled by a motion-triggered switch placed in parallel to the energy storage unit [193]. The undesirable small output current from TENGs can also be enhanced, e.g., using sliding micro-grating [141], radial-arrayed [142] or multi-layered stacked structures [194].
(iii) Durability and stability—Stable and reliable materials and devices are required for real life applications, which could constitute a problem mainly in the case of high-friction lateral-sliding TENGs. More durable composite structures must be investigated for TENGs or using a conjunction of working modes. Strategies already tested have included mechanically switchable structures with automatic transition between contact (triboelectric charge build-up) and non-contact working states [195] and low-friction free-standing structures with intermediate rolling rods [149]. Structures with flexible hair brushes have been used to greatly reduce the operation friction resistance, enhance the device durability and solve the charge dissipation problem [76]. The friction forces of TENGs fabricated by PTFE were shown to be lower than those with PVC and FEP under the same pressure, namely in wind energy harvesting excited by the movement of high-speed rail vehicles [196]. Thus, based on the reasonable selection of dielectric materials with minimal friction coefficient, the results highlight that the friction force of a double-layer PTFE/Kapton elastic rotation TENG was greatly reduced, the energy harvesting efficiency was doubled and its durability increased by 4 times [196]. Another effective strategy to simultaneously enhance the power density and durability of sliding-mode TENGs is via liquid lubrication interface [197]. Operation in a squalene solution has been shown to reduce the friction force, suppress the interfacial electrostatic breakdown and decrease the charge loss by more than 50% [197].
(iv) Packaging—The performance of the nanogenerators, especially TENGs, sensibly depends on the moisture and temperature. Thus, good packaging methods for devices with moving parts are required to guarantee a thorough water sealing [65], while raising the working temperature of the devices could also prove useful [198]. Humidity-resistive TENGs were also fabricated making use of metal organic framework composites to increase the electron-trapping capacity and dielectric constant [123].
(v) Energy storage—Energy storage of irregular electrical outputs is required. Improvements must be carried out regarding the leakage problem in supercapacitors and new designs of Li-ion batteries should be tested [199].
(vi) Operation input—Wider bandwidths of input frequencies and amplitudes are valuable. This could be achieved, e.g., by periodic tuning of the resonant frequency, mechanical stoppers, nonlinear springs or bi-stable structures [200]. Low frequencies and amplitudes of excitation are associated with the low input powers and predominance of velocity independent Coulomb’s mechanical dry friction. Some solutions to overcome this problem might include the use of flexible brushes as contact materials [76], rational selection of low-friction dielectric materials [196] and interface liquid lubrication [197].
(vii) Understanding of the conversion mechanism—Obtaining a better understanding of the triboelectric effect and its relation to the environmental conditions could be useful to optimize the performance of the harvesters. Experimental studies using, e.g., atomic force microscopy and Kelvin probe microscopy [112, 113] and theoretical first principle quantum physical studies are still lacking. New techniques to accurately measure the surface charge density and its relation to the dielectric properties and surface morphology of the materials would be welcome. An updated quantitative standard triboelectric series could be important for material choice guidance. New computational models to predict the electromechanical output for realistic irregular tri-dimensional motions of combined hybrid harvesters should also be developed.
(viii) Quantitative standardization—Quantitative experimental standards should be employed for comparing and calibrating the performance of developed E-TENG prototypes [118], since testing conditions vary widely in the literature. Reporting of average power or conversion efficiency values should also be preferred to maximum instantaneous peak power due its high sensitivity to the time form of the input mechanical excitation. Values of the input displacement, acceleration or force amplitudes should be provided.
(ix) System integration—Rational designs should be developed for system integration with different kinds of vibration environments. Biomechanical and hydrodynamic fluid flow studies could be important [78, 107]. Economic studies should be carried out to estimate the maintenance and production costs of hybrid nanogenerators to evaluate their competitiveness in the energy market. The possible impact of relatively large stray magnetic and electric fields associated with EMGs and TENGs, respectively, should also be addressed.
(x) Miniaturization—Miniaturization of E-TENGs for the integration with wearable equipment or incorporation within innovative implantable bioelectronic medical devices should be explored [201]. Significant output powers from biomechanical sources and the ability to work in biological environment are relevant requirements for future advanced applications of miniaturized E-TENGs. Small-scale individual generators are expected to provide small output powers, although multiple arrays of such devices can theoretically yield improved results. Shape-adaptive TENGs made of elastomeric materials and mounted under shoes or weaved into clothing for powering wearable electronics have already been developed [202, 203]. Fiber-based TENGs for wearable power sources that could be woven into fabric have also been tested [204, 205]. EMGs are expected to suffer from poor volume downscaling with rapidly dropping output powers and increasing resonance frequencies. Their characteristic low output voltages can also result in no output power because of the certain voltage level required to polarize diodes in the rectification circuit. Assembling small-scale coils and magnets might also prove challenging. TENGs should have a better volume scaling behavior although problems related to charge leakage due to imperfect insulation and dielectric breakdown due to thinner dielectrics are anticipated. The predominance of surface effects, including stray electric fields at the edges and corresponding parasitic capacitances, is also expected.
(xi) Large-scale manufacturing—While large-scale EMGs are easier to manufacture, as only conventional machining technology is required, large-scale triboelectric generators require special attention, because upscaling of the laboratory production techniques such as pulsed laser deposition (PLD) or atomic layer deposition (ALD) to a mass production level is difficult. Moreover, standard techniques reported in the literature to boost the performance of TENGs involve nano/micro-patterning of the electrodes, which is complex, tedious and expensive. The solutions for the large-scale patterning could be soft lithography as well as surface treatment processes, including different kinds of plasma treatment processes and chemical synthesis methods suitable for large-scale manufacturing [206]. For the layer deposition, several techniques have been used such as s flexible printed circuit manufacture, inkjet printing, screen printing and roll-to-roll patterning [206]. The choice of the method is determined by the materials used and type of the device to be manufactured.
(xii) Multi-energy hybrid cells—Hybrid cells to simultaneously harvest energy from multiple available types of environmental energy, including solar, thermal and mechanical, will most likely be very useful in future [71].

These questions raise many opportunities in the development of hybrid triboelectric-electromagnetic nanogenerators for researchers and entrepreneurs across all fields. We thus expect this field to experience a rapid growth in the next decade.
